# Development of an efficient deep learning model for arrhythmia detection from consumer smartwatches

**DOI:** 10.3389/frai.2026.1882739

**Published:** 2026-09-28

**Authors:** Dan-Cristian George, Mihai-Alexandru Hulubeac, Ştefan-Lucian Grămadă, Zoltán Bálint, Laura Dioşan

**Affiliations:** 1 Department of Computer Science, Faculty of Mathematics and Computer Science, Babeş-Bolyai University, Cluj-Napoca, Romania; 2 Department of Biomedical Physics, Faculty of Physics, Babeş-Bolyai University, Cluj-Napoca, Romania

**Keywords:** arrhythmia detection, atrial fibrillation detection, deep learning, heart rhythm monitoring, photoplethysmography (PPG), recurrent neural networks, smartwatch monitoring, wearable devices

## Abstract

**Introduction:**

Cardiac arrhythmias often progress without clear symptoms and may remain undetected until severe complications occur. Continuous monitoring through consumer smartwatches offers a scalable and non-invasive opportunity for real-time rhythm screening in free-living conditions. However, wrist-based photoplethysmography (PPG) signals are highly susceptible to motion artifacts and inter-subject variability, limiting traditional signal-processing techniques. In this work, we compare classical machine learning and deep learning approaches for multi-class arrhythmia classification from real-life smartwatch PPG and accelerometer recordings collected in the Pulsewatch clinical trial. Three rhythm categories are considered: normal sinus rhythm (NSR), atrial fibrillation (AF), and premature atrial or ventricular contractions (PAC/PVC).

**Methods:**

The evaluated approaches include a Random Forest classifier based on engineered temporal and morphological features, together with two end-to-end architectures, CNN–GRU and CNN–BiLSTM, designed to learn temporal patterns directly from raw two-channel smartwatch waveforms while addressing motion artifacts, sensor noise, and cross-subject generalization.

**Results:**

Under two-fold subject-independent cross-validation, the Random Forest achieved the highest average accuracy among the proposed methods (91.22%), together with the best macro precision (85.89%), macro F_1_ score (84.17%), and macro ROC-AUC (97.03%). The CNN–BiLSTM achieved the highest macro recall (87.37%), indicating stronger sensitivity to arrhythmic classes, while the CNN–GRU remained competitive with a macro recall of 86.85% and a macro F_1_ score of 83.13%.

**Discussion:**

Compared with the Pulsewatch baselines reported by Han et al., the proposed methods improved the available macro-level metrics, with the Random Forest obtaining the strongest macro precision and macro F_1_ score and the CNN–BiLSTM the strongest macro recall, indicating improved class-balanced recognition of clinically relevant arrhythmic events. These results show that feature-based models remain highly competitive for smartwatch-based arrhythmia detection, achieving the strongest overall and macro F_1_ performance among the proposed methods, while hybrid convolutional–recurrent architectures provide increased sensitivity to minority rhythm classes. Deployment-oriented profiling under an identical single-core benchmark showed a mean end-to-end inference time of 33.08 ms for the Random Forest pipeline and 51.87 ms for the CNN–BiLSTM model on a 30-s segment, supporting near-real-time rhythm screening.

**Conclusion:**

Overall, this work highlights the trade-offs between the analyzed approaches in terms of accuracy, class-balanced performance, computational complexity, and deployment feasibility for wearable cardiac monitoring systems.

## 1 Introduction

Cardiovascular diseases remain the leading cause of mortality worldwide, accounting for approximately 19.8 million deaths in 2022 and representing about 32% of all global deaths, while many clinically significant arrhythmias may occur intermittently or remain asymptomatic (World Health Organization, 2025; Roth et al., 2020; Hindricks et al., 2021). Early detection is therefore essential, yet conventional diagnostic tools such as electrocardiography are typically applied only after clinical suspicion arises.

Continuous monitoring through wearable devices offers a complementary paradigm, enabling long-term observation of cardiac rhythm in everyday life. This potential has been demonstrated across both clinical validation studies and large-scale population deployments. Mannhart et al. (2023) performed a prospective clinical validation of five consumer wearable devices against 12-lead ECG, reporting sensitivities of up to 85% for atrial fibrillation detection in real-world patients. At population scale, studies such as the Apple Heart Study by Perez et al. (2019) and the Fitbit Heart Study by Lubitz et al. (2022) showed that wearable PPG-based algorithms can reliably identify previously undiagnosed atrial fibrillation with high positive predictive value in ambulatory settings, while the Huawei Heart Study further demonstrated the effectiveness of both population-wide and targeted screening strategies using wearable devices (Guo et al., 2020). From a methodological perspective, Pereira et al. (2020) provided a comprehensive review of photoplethysmography-based atrial fibrillation detection approaches, highlighting the feasibility of continuous, non-invasive rhythm monitoring, while more recent work by Abdelrazik et al. (2025) emphasized the growing clinical relevance of wearable systems and the increasing integration of artificial intelligence techniques.

Among the arrhythmias amenable to wearable monitoring, atrial fibrillation (AF) is of foremost clinical concern. AF is the most prevalent sustained cardiac arrhythmia, affecting an estimated 37.5 million individuals worldwide and it is associated with a fivefold increased risk of ischemic stroke, heightened incidence of heart failure, and substantial all-cause mortality (Hindricks et al., 2021). Clinically, AF is characterized by disorganized atrial electrical activity producing an irregular ventricular response, absence of distinct P waves, and variable RR intervals (Hindricks et al., 2021). According to the 2020 European Society of Cardiology guidelines, AF is stratified into four clinical patterns based on episode duration and spontaneous termination: *paroxysmal* AF (self-terminating, typically within 48 h), *persistent* AF (sustained beyond 7 days or requiring pharmacological or electrical cardioversion), *long-standing persistent* AF (continuous for more than 12 months), and *permanent* AF (in which sinus rhythm restoration is no longer pursued) (Hindricks et al., 2021). Diagnosis is conventionally established through standard 12-lead electrocardiography or ambulatory Holter monitoring; however, the episodic and often asymptomatic nature of paroxysmal AF means that brief clinical recordings frequently fail to capture relevant events, making prolonged monitoring essential for reliable detection (Hindricks et al., 2021; Perez et al., 2019; Lubitz et al., 2022). These diagnostic limitations motivate the exploration of continuous wrist-based photoplethysmography as a scalable, non-invasive tool for long-term cardiac rhythm surveillance (Pereira et al., 2020).

Modern smartwatches acquire photoplethysmography signals at the wrist and can record complementary motion information through embedded accelerometers. This combination enables large-scale, non-invasive monitoring of heart rhythm outside clinical environments (Charlton et al., 2022; Castaneda et al., 2018). However, wrist-based photoplethysmography signals are inherently noisy and strongly influenced by motion artifacts, sensor placement, physiological variability, and environmental factors (Lee et al., 2020; Zhang et al., 2019). These characteristics make arrhythmia detection from smartwatch data a challenging pattern-recognition problem that requires robust and generalizable computational methods (Bashar et al., 2019).

Traditional feature-engineering approaches rely on manually designed heart rate variability metrics and rule-based signal processing (Task Force of the European Society of Cardiology the North American Society of Pacing Electrophysiology, 1996; Oh et al., 2018; Wang et al., 2020). While interpretable, such methods may struggle to capture complex temporal dependencies and subtle morphological variations in real-world signals. In contrast, deep learning architectures can automatically learn hierarchical representations directly from raw time-series data and model long-range temporal dynamics (Ebrahimi et al., 2020). Recurrent neural networks such as gated recurrent units and long short-term memory networks are particularly suitable for sequential physiological data, as they can capture dependencies across multiple cardiac cycles (Ansari et al., 2023). Recent advances further demonstrate that wearable data can capture broader physiological patterns beyond cardiac rhythm, enabling the prediction of systemic health conditions through data-driven approaches. Metwally et al. (2026) introduced a large-scale study (WEAR-ME, 1,165 participants) combining smartwatch time-series data with routine blood biomarkers to predict insulin resistance, a key precursor of type 2 diabetes. Their multimodal deep learning model achieved an ROC-AUC of 0.80, highlighting that continuous wearable signals can reveal early metabolic dysfunction not detectable through conventional clinical snapshots (Metwally et al., 2026). Similarly, recent work on wearable-based glucose prediction has shown that non-invasive sensor data can be used to estimate glycemic dynamics using machine learning models, reducing reliance on continuous glucose monitoring devices (Huang et al., 2025). More broadly, surveys of artificial intelligence applications in wearable health systems indicate that multimodal approaches integrating physiological signals with clinical data can support prediction, monitoring, and personalized management of chronic diseases such as diabetes and cardiovascular disorders (Fraser et al., 2025).

Beyond predictive performance, a critical challenge in smartwatch-based arrhythmia detection is the feasibility of real-time inference under strict computational and energy constraints. Early work such as DeepBeat by Torres-Soto and Ashley (2020) demonstrated that deep learning models can be designed for real-time atrial fibrillation detection directly from wearable photoplethysmography signals, while simultaneously handling signal quality estimation through a multi-task architecture. However, subsequent studies have highlighted that standard deep neural networks are often too computationally demanding for direct deployment on smartwatch hardware, requiring either cloud-based inference or model simplification (Bashar et al., 2019). Recent research has therefore focused on designing lightweight and efficient architectures tailored for embedded and wearable environments. Maekynen et al. (2024) proposed compressed deep learning models with attention mechanisms that reduce computational cost while preserving discriminative performance for atrial fibrillation detection. Similarly, Tang et al. (2025) introduced a low-complexity neural architecture optimized for real-time arrhythmia detection on embedded platforms, leveraging depthwise separable convolutions to reduce parameter count and inference latency. In parallel, studies on continuous monitoring using smartwatch PPG signals have shown that deep neural networks can process a larger proportion of noisy real-world data compared to traditional signal-processing pipelines, enabling more reliable near-continuous rhythm analysis in free-living conditions (Antiperovitch et al., 2024). These developments highlight a key trade-off in wearable health systems between model complexity, robustness to motion artifacts, and real-time deployability, motivating the exploration of architectures that balance predictive performance with computational efficiency.

In this work, we conduct a systematic comparative study of two modeling strategies for smartwatch-based arrhythmia detection, namely a Random Forest classifier based on engineered temporal and motion-related features and a recurrent neural network based on gated recurrent units. In addition, we propose a hybrid convolutional-recurrent architecture based on a CNN–BiLSTM model, designed to learn discriminative temporal patterns directly from multimodal smartwatch signals. All approaches were evaluated on the Pulsewatch dataset, which contains real-world smartwatch photoplethysmography and accelerometer recordings aligned with electrocardiogram-derived rhythm annotations from the Pulsewatch clinical trial (ClinicalTrials.gov identifier: NCT03761394) (Ding et al., 2023). Importantly, evaluation was performed using a two-fold subject-independent cross-validation protocol to assess generalization to unseen individuals. In each direction, models were trained and validated on one fold and tested on the opposite fold, whose subjects were not used during training or model selection.

Relative to the Pulsewatch-based framework proposed by Han et al. (2025), which employs a computationally efficient multimodal one-dimensional bidirectional gated recurrent unit (1D Bi-GRU) architecture operating on smartwatch photoplethysmography (PPG), heart rate, and accelerometer signals for three-class arrhythmia classification, the present work provides a complementary analysis of model design choices for smartwatch-based arrhythmia detection. Han et al. demonstrated that recurrent neural networks can achieve strong performance on Pulsewatch when using PPG together with heart-rate-derived and motion-related inputs. In this manuscript, the focus is placed on comparing modeling strategies under the same subject-independent protocol, using both an interpretable feature-based pipeline and end-to-end architectures that operate directly on raw two-channel PPG and accelerometer waveforms.

At the architectural level, the CNN–GRU model keeps the convolutional–recurrent structure suitable for temporal smartwatch signals, but removes engineered heart-rate inputs and adds temporal attention to weight informative regions within each 30-s segment. The CNN–BiLSTM model follows the same raw multimodal input setting and replaces the GRU block with a bidirectional LSTM block, aiming to better capture longer temporal dependencies and subtle rhythm irregularities. This comparison allows the contribution of each modeling choice to be evaluated in terms of class-balanced performance, interpretability, computational cost, and deployment feasibility. The added value is quantified against Pulsewatch baselines reported by Han et al., with particular attention to macro-level performance, class-wise detection of atrial fibrillation and PAC/PVC events, and deployment-oriented feasibility.

## 2 Related work

Han et al. (2025) proposed a multiclass arrhythmia classification framework based on smartwatch-acquired photoplethysmography signals collected in real-life conditions. Their study relies on data from the Pulsewatch clinical trial (ClinicalTrials.gov identifier: NCT03761394), in which a total of 90 participants were randomized into the intervention arm of phase 1 and 57 participants into the phase 2 intervention group. Participants wore a smartwatch together with a chest ECG patch for approximately 14 days, with signals recorded at 50 Hz. From this cohort, 116,313 non-overlapping 30-s segments from 72 subjects were used under a subject-independent two-fold cross-validation protocol, covering normal sinus rhythm, atrial fibrillation, and premature atrial or ventricular contractions. A detailed overview of the dataset distribution across folds and rhythm classes is provided in Table 1.

**Table 1:** Pulsewatch dataset distribution across the two subject-independent folds and rhythm classes.

Subjects (non-exclusive counts)				
Fold	NSR	AF	PAC/PVC	Unique subjects
Fold-1	18	17	19	36
Fold-2	18	18	19	36
Total	36	35	38	72
**Segments (mutually exclusive counts)**				
**Fold**	**NSR**	**AF**	**PAC/PVC**	**Total segments**
Fold-1	39 356	12 265	6 697	58 318
Fold-2	39 363	12 290	6 342	57 995
Total	78 719	24 555	13 039	116 313

Subject counts per rhythm class are not mutually exclusive; a subject is counted in a class if it contributes at least one segment belonging to that rhythm category.

The authors applied a sixth-order Butterworth band-pass filter between 0.5 and 20 Hz to the PPG signals, followed by normalization and heart rate extraction via peak detection, while excluding segments with more than 5 s of motion noise. A lightweight one-dimensional bidirectional GRU architecture was trained using multimodal inputs, including the filtered PPG waveform, heart rate, accelerometer magnitude, and a magnified heart rate representation. The best-performing configuration achieved approximately 97.31% accuracy for atrial fibrillation detection and 83.52% sensitivity for PAC/PVC classification. This four-channel configuration contained around 120,224 parameters and required approximately 0.89 GFLOPs, being significantly smaller and faster than comparison models. Furthermore, the approach demonstrated strong external generalization, reaching macro ROC-AUC values of about 96.22% and 94.17% on two independent PPG datasets.

While prevailing literature on Atrial Fibrillation (AF) detection has traditionally centered on ECG-based analysis through handcrafted features derived from heart rate variability (HRV) and waveform morphology, recent advancements suggest that photoplethysmography (PPG)-based solutions offer a robust alternative for large-scale screening. Due to their non-invasive nature and compatibility with wearable devices such as smartwatches, PPG-based Random Forest (RF) models provide a significant practical advantage over traditional ECG methods, enabling continuous, real-time monitoring. These models have demonstrated remarkable empirical performance, achieving accuracies of 90% on the University of Mississippi Medical Center (UMMC) dataset (*n* = 216) and 97% on the Medical Information Mart for Intensive Care (MIMIC)-III dataset (*n* = 2, 134) (Talukdar et al., 2023).

A fundamental distinction between the RF classifier and end-to-end deep learning architectures lies in the feature-dependent nature of the former. Unlike deep learning models that process raw PPG waveforms, the RF approach requires a dedicated feature engineering stage. Following the methodologies established in Eerikäinen et al. (2019), we utilized an extensive feature set categorized as follows:

- **Inter-Pulse Interval (IPI) features**: This set includes Shannon Entropy (ShEn), Normalized Root Mean Square of Successive Differences (nRMSSD), *pNN*40, *pNN*70, Sample Entropy (*SampEn*), and the Coefficient of Sample Entropy (*CoSEn*). Additionally, the Mean Markov scores for AF/AFL (derived from both IPIs and Peak-to-Peak Interval sequences), along with the maximum, minimum, median, and standard deviation of IPIs, were incorporated.
- **PPG waveform morphology features**: To capture the signal's shape, we extracted Kurtosis (*K*), Hjorth mobility (*H*_1_), Hjorth complexity (*H*_2_), Spectral Purity Index (*SPI*), and Spectral Entropy (*SE*). Pulse amplitude metrics, including maximum, minimum, mean, and standard deviation, were also calculated.
- **Accelerometer-based features**: To account for physical activity, the standard deviation and maximum absolute value of the accelerometer magnitude were utilized.

To further enhance the multi-class discrimination of Premature Atrial Contractions (PAC) and Premature Ventricular Contractions (PVC) from Normal Sinus Rhythm (NSR) and AF, we integrated Poincaré plot analysis as proposed by Han et al. (2020). This method maps the inter-beat interval sequences into a geometric representation, allowing for the extraction of six specialized features that characterize non-linear cardiac dynamics:

- ***SD_1_***: The standard deviation of points perpendicular to the identity line, representing short-term heart rate variability.
- ***SD_2_***: The standard deviation along the identity line, capturing long-term variability.
- ***SD_ratio_***: The ratio *SD*_1_/*SD*_2_, quantifying the relationship between short- and long-term fluctuations.
- ***Kite_count_***: A morphological metric that counts "kite-shaped" structures in the plot, serving as specific indicators of ectopic beats (PAC/PVC).
- ***Kite_std_deg_***: The standard deviation of the angles formed by vectors in the kite model, providing a measure of geometric consistency.
- ***Zero_ratio_***: The proportion of points concentrated in the central region of the plot (variation < 5 bpm), indicating the stability of the sinus rhythm.

Recognizing the high sensitivity of PPG sensors to motion, the accelerometer magnitude was also employed to filter motion artifacts and ensure signal integrity, following the noise reduction techniques described in Comtois et al. (2007).

Faust et al. (2018) proposed a deep learning system for automated atrial fibrillation detection from heart rate signals, applying a bidirectional long short-term memory network directly to sliding windows of RR interval sequences without any intermediate feature extraction stage. The input data was partitioned into blocks of 100 consecutive beat intervals and fed directly into the deep bidirectional LSTM, enabling the network to implicitly learn the temporal irregularity patterns characteristic of AF. The system was evaluated on the MIT-BIH Atrial Fibrillation Database, achieving 98.51% accuracy under ten-fold cross-validation and 99.77% under blind-fold validation. Although this approach demonstrates the strong discriminative power of bidirectional LSTM models for binary AF vs. normal sinus rhythm classification, it operates on derived beat interval sequences from clean, stationary ECG recordings in a controlled clinical setting. In contrast, our CNN–BiLSTM architecture directly processes raw two-channel wrist photoplethysmography and accelerometer waveforms in a three-class arrhythmia regime, addressing the additional complexities of motion artifacts, consumer-grade sensor noise, and generalization to unseen subjects under a strict subject-independent evaluation protocol.

Petmezas et al. (2021) proposed a hybrid CNN–LSTM architecture for automated atrial fibrillation detection on imbalanced ECG datasets. In their model, a one-dimensional convolutional network first extracts discriminative morphological features from raw ECG segments, which are then passed to an LSTM layer to capture temporal dynamics and long-range dependencies across the sequence. To address the inherent class imbalance present in the MIT-BIH Atrial Fibrillation Database, the model employs focal loss in place of standard cross-entropy, penalizing easy examples less and focusing training on hard-to-classify minority instances. The system performs four-class rhythm classification covering normal rhythm, atrial fibrillation, atrial flutter, and AV junctional rhythm, achieving a sensitivity of 97.87% and a specificity of 99.29% under 10-fold cross-validation. While this CNN–LSTM pipeline demonstrates strong discriminative performance for multi-class arrhythmia detection from clinical ECG signals, it relies on single-modality, high-fidelity recordings in a controlled laboratory setting. Our work adopts a comparable hybrid convolutional–bidirectional LSTM strategy but targets a more challenging deployment scenario, processing noisy two-channel photoplethysmography and accelerometer waveforms acquired by consumer smartwatches in free-living conditions and generalizing across unseen subjects through a strict subject-independent evaluation protocol.

## 3 Dataset description

The dataset used for training and evaluating the arrhythmia classification models in this work is the publicly referenced Pulsewatch clinical trial (ClinicalTrials.gov identifier: NCT03761394) (Han et al., 2025). The Pulsewatch dataset was collected as part of an NIH-funded study designed to assess the feasibility of using smartwatch photoplethysmography (PPG) and accelerometer signals for heart rhythm monitoring in real-world ambulatory conditions. Participants aged 50 years or older with a documented history of ischemic stroke were recruited for continuous monitoring using wearable devices.

Each participant concurrently wore a smartwatch (either a Samsung Gear S3 or a Samsung Galaxy Watch 3) and a chest-mounted electrocardiogram (ECG) patch. The smartwatch recorded single-lead PPG at 50 Hz along with accelerometer data derived from a tri-axial sensor for motion information, while the ECG patch provided a high-fidelity gold-standard reference at 250 Hz. In the provided dataset, the accelerometer signal is available as a single scalar magnitude per time step, representing the overall motion intensity. ECG labels were used to adjudicate the corresponding smartwatch PPG segments into rhythm categories. Motion artifact detection and quality filtering were applied prior to rhythm annotation, retaining only 30-s segments containing no more than five seconds of motion noise for analysis.

The dataset is partitioned into two subject-independent folds. In total, 72 distinct subjects contributed usable data, with each fold comprising 36 subjects. Within each fold, the data is further divided into training (80%), validation (10%), and held-out test (10%) subsets, ensuring that samples from the same subject do not appear across these subsets. Across both folds there are 116,313 labeled 30-s PPG segments available for classification, distributed among normal sinus rhythm (NSR), atrial fibrillation (AF), and premature atrial or ventricular contraction (PAC/PVC) classes.

Table 1 summarizes the distribution of both subjects and segments across folds and rhythm categories. At the segment level, the dataset exhibits a pronounced class imbalance, with normal sinus rhythm representing the majority class (67.68%), followed by atrial fibrillation (21.11%) and a smaller proportion of PAC/PVC segments (11.21%). At the subject level, the class-wise counts are not mutually exclusive, as a single subject may contribute segments belonging to multiple rhythm categories. Consequently, a subject is counted in a given class if it provides at least one segment labeled with that rhythm, which explains why the per-class subject counts exceed the number of unique individuals within each fold.

These segments were derived from continuous recordings over an extended monitoring period, providing motion-artifact prone, real-world PPG and accelerometer data representative of free-living conditions. The dataset's real-world nature and multi-class rhythm annotations make it a valuable resource for developing and benchmarking wearable arrhythmia classification algorithms that operate without reliance on ECG.

The dataset contains smartwatch PPG recordings sampled at 50 Hz, accelerometer magnitude signals sampled at 50 Hz, and ECG-derived reference rhythm annotations sampled at 250 Hz, aligned to 30-s labeled segments, together with subject- and segment-level metadata.

This makes Pulsewatch particularly suitable for evaluating smartwatch-based arrhythmia detection models under realistic free-living conditions, where motion corruption, inter-subject variability, and class imbalance are expected.

## 4 Approach

### 4.1 General framework

To ensure a consistent and fair comparison between classical machine learning and end-to-end deep learning approaches, we define a unified framework for multimodal arrhythmia detection from smartwatch signals. The framework specifies the common input representation, prediction task, training and evaluation protocol, and evaluation metrics shared across all models, while allowing each modeling paradigm to use an appropriate data processing and representation-learning strategy.

#### 4.1.1 Multimodal input acquisition

All approaches operate on the Pulsewatch dataset described in Section 3. The input consists of synchronized photoplethysmography (PPG) and accelerometer (ACC) signals acquired from consumer smartwatches at a sampling frequency of 50 Hz. The dataset provides pre-segmented 30-s windows, each corresponding to 1,500 time steps per modality. In the provided dataset, the accelerometer signal is available as a single scalar value per time step, representing overall motion intensity. Consequently, each segment is represented as a two-channel time series of shape 2 × 1, 500, corresponding to PPG and ACC signals, respectively.

#### 4.1.2 Output prediction

All models perform three-class classification corresponding to the rhythm categories defined in the Pulsewatch dataset: normal sinus rhythm (NSR), atrial fibrillation (AF), and premature atrial or ventricular contractions (PAC/PVC). In addition to class predictions, certain models provide interpretability outputs, such as feature importance scores in the case of the Random Forest classifier or temporal attention maps in the case of deep learning architectures.

#### 4.1.3 Training and evaluation protocol

All models are trained and evaluated using the predefined two-fold subject-independent partition of the Pulsewatch dataset described in Section 3. In the first cross-validation direction, models are trained using the training subset of Fold 1, validated on the validation subset of Fold 1, and tested on Fold 2. In the reverse direction, models are trained using the training subset of Fold 2, validated on the validation subset of Fold 2, and tested on Fold 1. Since the two folds contain disjoint subject groups, each test fold consists entirely of subjects that were not used during training, validation, hyperparameter selection, early stopping, or model selection in that evaluation direction.

The held-out test subset within the training fold is not included in the main reported evaluation, in order to keep the analysis focused on subject-independent cross-fold generalization. The final reported performance corresponds to the average of the metrics obtained across the two subject-independent test directions, Fold 1 → Fold 2 and Fold 2 → Fold 1. This protocol provides a direct assessment of cross-subject generalization and aligns the evaluation with previous Pulsewatch-based studies using two-fold subject-independent validation.

Both folds exhibit pronounced class imbalance at the segment level, with normal sinus rhythm (NSR) representing the majority class, followed by atrial fibrillation (AF), and premature atrial or ventricular contractions (PAC/PVC). This imbalance reflects realistic clinical distributions but introduces challenges for minority class detection. To mitigate this effect, class imbalance is addressed through class weighting or oversampling strategies, depending on the model. These operations are applied only within the training subset of the corresponding cross-validation direction, while validation and test data retain their original class distributions. This prevents information leakage from the opposite test fold and ensures that the reported results reflect performance under the original class distribution.

#### 4.1.4 Evaluation metrics

Performance is evaluated using accuracy, macro precision, macro recall, macro F_1_ score, and macro ROC-AUC, ensuring a comprehensive assessment of both overall discrimination and class-balanced behavior. Let *C* denote the number of classes and, for each class *i*, let TP_*i*_, FP_*i*_, TN_*i*_, and FN_*i*_ denote the number of true positives, false positives, true negatives, and false negatives computed in a one-vs.-rest manner.

Accuracy is defined in Equation 1, where N denotes the total number of evaluated segments:


$$\begin{array}{c} \text{Accuracy}=\frac{\overset{C}{\underset{i=1}{\sum }}{\text{TP}}_{i}}{N} \end{array}$$ (1)

Per-class precision and recall are defined in Equation 2:


$$\begin{array}{c} {\text{Precision}}_{i}=\frac{{\text{TP}}_{i}}{{\text{TP}}_{i}+{\text{FP}}_{i}},\;{\text{Recall}}_{i}=\frac{{\text{TP}}_{i}}{{\text{TP}}_{i}+{\text{FN}}_{i}} \end{array}$$ (2)

Macro-averaged precision and recall are computed according to Equation 3:


$$\begin{array}{ccc} \text{Precision Macro} & = & \frac{1}{C}\overset{C}{\underset{i=1}{\sum }}{\text{Precision}}_{i}, \end{array}$$

$$\begin{array}{ccc} \text{Recall Macro} & = & \frac{1}{C}\overset{C}{\underset{i=1}{\sum }}{\text{Recall}}_{i} \end{array}$$ (3)

The macro _1_ score, defined as the average of the per-class F1 scores, is computed according to Equation 4.


$$\begin{array}{c} {\text{MacroF}}_{1}=\frac{1}{C}\overset{C}{\underset{i=1}{\sum }}\frac{2\cdot {\text{Precision}}_{i}\cdot {\text{Recall}}_{i}}{{\text{Precision}}_{i}+{\text{Recall}}_{i}} \end{array}$$ (4)

The macro ROC-AUC is computed by averaging the area under the receiver operating characteristic curve across all classes in a one-vs.-rest manner (Fawcett, 2006). These metrics are widely used in imbalanced multi-class classification settings, as they provide a balanced evaluation across all classes regardless of their frequency.

#### 4.1.5 Models comparison

The investigated models are compared as complete modeling pipelines rather than as isolated classifiers operating on identical intermediate representations. The Random Forest pipeline intentionally includes explicit signal preprocessing and handcrafted feature extraction, reflecting a classical domain-knowledge-driven approach to wearable arrhythmia detection. In contrast, the CNN–GRU and CNN–BiLSTM pipelines use minimally processed two-channel smartwatch waveforms and rely on the neural architectures to learn discriminative temporal representations directly from the data. This design enables a comparison between feature-based and end-to-end modeling paradigms under the same dataset, cross-validation protocol, and evaluation metrics.

Because the Random Forest relies on handcrafted features and runs only on the CPU, whereas neural models can normally benefit from hardware acceleration, a dedicated benchmark was built to evaluate the deployment profile of each pipeline under identical conditions. All models expose the same inference interface: they receive a single 30-s smartwatch segment and produce one class label, NSR, AF, or PAC/PVC. Each model keeps its own preprocessing pipeline behind this interface, with preprocessing and prediction timed separately and combined in the reported end-to-end latency.

All benchmark experiments ran on a Lenovo Legion Pro 5 16IAX10H with an Intel Core Ultra 9 275HX, under Linux CachyOS, kernel 7.1.3-1-cachyos, Python 3.11.15, PyTorch 2.6.0 and scikit-learn 1.8.0. To keep the comparison fair, CUDA was disabled so that every model ran on the CPU. The process was pinned to a single core, core 0, and every math backend was restricted to one thread (OpenMP, MKL and OpenBLAS set to one, PyTorch single-threaded, and the Random Forest with n_jobs = 1), removing any advantage of multi-threading. Turbo Boost was disabled (no_turbo = 1) and the frequency governor fixed to *performance*, so the clock stays constant and latencies are reproducible.

For each profiled model, 900 segments were randomly selected using a fixed seed of 42 for reproducibility, with a balanced subset of 300 segments per class. Only one model was loaded per run, and the 900 segments were loaded into RAM before timing, so disk I/O was excluded. After warm-up inferences, each run performed 10 repetitions, with one repetition classifying all 900 segments, yielding 9,000 timed inferences per model. For every inference, preprocessing time and prediction time were recorded separately and written to a CSV file for later analysis. Memory was characterized through the Resident Set Size (RSS), sampled immediately before and after loading the model. Their difference estimates the model memory footprint, while the peak RSS captures the maximum total memory held by the process during the timed run.

#### 4.1.6 Feature-based approach data handling

For the feature-based approach, each segment is transformed into a fixed-length feature vector derived from temporal, morphological, and motion-related characteristics. This approach applies explicit signal processing, including band-pass filtering and accelerometer-informed motion artifact removal, prior to feature extraction.

Feature extraction is performed independently for each segment, ensuring that no information is shared across training, validation, or test data. Class imbalance is handled through class weighting in the Random Forest classifier, with weights estimated only from the training subset of the corresponding cross-validation direction. Within this modeling paradigm, handcrafted descriptors capturing heart rate variability, waveform morphology, and motion characteristics are extracted and used as input to a classical machine learning classifier. Noise robustness is addressed explicitly through accelerometer-informed filtering and the design of motion-aware features.

#### 4.1.7 Deep learning-based data handling

For the end-to-end deep learning models, the two-channel time-series representation of shape 2 × 1, 500 is used directly as input. These models operate on minimally processed smartwatch signals and rely on convolutional and recurrent layers to learn discriminative temporal representations directly from raw multimodal data, without explicit feature engineering.

For each segment, the PPG channel is normalized independently using min–max scaling, while the accelerometer channel is normalized using a fixed physical range between 0 and 20 m/s^2^. This normalization is applied independently at segment level and does not require statistics estimated from validation or test data. To mitigate class imbalance during neural network training, random oversampling is applied only to the training subset of the corresponding cross-validation direction, with sampling applied to all classes except the majority class. Validation and test data are never oversampled and retain their original class distributions, reflecting the class imbalance expected under realistic deployment conditions.

Within this modeling paradigm, noise robustness is learned implicitly through convolutional filtering, recurrent temporal modeling, and attention-based temporal aggregation. This allows the CNN–GRU and CNN–BiLSTM models to process raw multimodal smartwatch waveforms directly and to learn rhythm-relevant temporal patterns without relying on handcrafted preprocessing or explicit denoising procedures.

### 4.2 Random Forest classifier

The main advantage of the Random Forest classifier lies in its interpretability and CPU-oriented execution. Compared with neural architectures, Random Forests can be trained using standard machine learning workflows and provide transparent feature-based decisions through feature importance and SHAP analysis. However, their deployment suitability depends strongly on ensemble size and tree depth, which directly affect storage, memory footprint, and inference cost.

To address the inherent sensitivity of PPG sensors to motion artifacts (MA), a filter described in Comtois et al. (2007) was applied on the PPG segments before the features were extracted. The motion artifacts are modeled using the magnitude of the three-axis accelerometer (ACC) signal, which serves as a correlated noise reference. The denoised signal, *s*_clean_(*n*), is obtained by subtracting the estimated motion component from the raw PPG observation, as expressed in Equation 5.


$$\begin{array}{c} s_{\text{clean}}\left(n\right)=s_{\text{raw}}\left(n\right)-\overset{L-1}{\underset{k=0}{\sum }}w_{k}\left(n\right)\cdot a_{\text{mag}}\left(n-k\right) \end{array}$$ (5)

where *a*_mag_ represents the Euclidean magnitude of the accelerometer, and *L* denotes the filter order, set to 16 as empirically optimized in the literature Comtois et al., (2007) to balance noise suppression and computational efficiency. Unlike static filters, the weight vector **w**(*n*) is updated iteratively for each sample *n* using the Least Mean Squares (LMS) algorithm, as shown in Equation 6.


$$\begin{array}{c} \text{w}\left(n+1\right)=\text{w}\left(n\right)+\mu \cdot s_{\text{clean}}\left(n\right)\cdot \text{x}\left(n\right) \end{array}$$ (6)

where μ is the step size, set to 0.016 as empirically determined in (Comtois et al. 2007), and $\text{x}\left(n\right)={\left[a_{\text{mag}}\left(n\right),a_{\text{mag}}\left(n-1\right),\ldots ,a_{\text{mag}}\left(n-L+1\right)\right]}^{⊤}$ is the vector of the *L* most recent accelerometer magnitude samples. The normalization of **x**(*n*) to unit variance prior to filtering ensures that the step size μ remains scale-independent across subjects with differing activity levels.

Furthermore, the LMS stage is preceded by a zero-phase fourth-order Butterworth bandpass filter applied in the range [0.5, 8.0] Hz, which removes DC drift and baseline wander while preserving the cardiac morphology of the PPG signal.

#### 4.2.1 Feature extraction

Following preprocessing, a fixed-length feature vector of 29 scalar descriptors is extracted from each 30-s segment. The features are organized into four groups:

##### 4.2.1.1 Inter-Peak Interval (IPI) features

Peaks are detected using a minimum inter-peak distance of 0.4s and a height threshold at the 25th percentile of the signal amplitude. The sequence of inter-peak intervals {*ipi*_*k*_} is then used to compute 12 features capturing heart rate variability (HRV):

- **Shannon Entropy (ShEn):** The normalized entropy of the empirical IPI histogram, quantifying the irregularity of the rhythm.
- **nRMSSD:** The root mean square of successive IPI differences, normalized by the mean IPI, providing a scale-independent measure of short-term variability.
- **pNN40, pNN70:** The proportion of successive IPI differences exceeding 40 ms and 70 ms, respectively, which are sensitive indicators of atrial fibrillation.
- **Sample Entropy (SampEn):** A regularity measure robust to noise, computed with embedding dimension *m* = 2 and tolerance *r* = 0.2σ_*ipi*_.
- **Conditional Entropy (CoSEn):** A normalized variant of SampEn that accounts for signal length and tolerance scale.
- **Markov IPI and Markov PPPIs:** Transition entropies computed from a three-state Markov chain fitted to the IPI and pulse-to-pulse interval sequences, respectively, capturing higher-order dependencies in rhythm dynamics.
- **IPI statistics:** Maximum, minimum, median, and standard deviation of the IPI sequence.

##### 4.2.1.2 PPG waveform features

Nine morphological and spectral features are computed directly from the (optionally cleaned) PPG signal:

- **Kurtosis:** The excess kurtosis of the signal amplitude distribution, sensitive to impulsive artifacts and waveform sharpness.
- **Hjorth mobility and complexity:** Parameters derived from the variance of the signal and its first and second derivatives, characterizing the frequency content and waveform complexity (Hjorth, 1970).
- **Spectral Peak Index (SPI):** The ratio of the dominant spectral power to the total power, estimated via Welch's method with a segment length of 256 samples.
- **Spectral Entropy (SpEn):** The Shannon entropy of the normalized power spectral density, measuring the distribution of power across frequencies.
- **Pulse amplitude statistics:** For each detected peak, the pulse amplitude is computed as the peak-to-trough excursion. The maximum, minimum, mean, and standard deviation of these amplitudes are extracted.

##### 4.2.1.3 Accelerometer features

Two features are derived from the ACC magnitude signal *a*_mag_: its standard deviation and absolute maximum. The standard deviation captures the overall intensity of motion activity throughout the segment, while the absolute maximum reflects the peak magnitude of any impulsive movement event.

##### 4.2.1.4 Poincaré plot features

Six features are derived from the Poincaré plot of successive heart rate differences, following the methodology of Han et al. (2020). The plot maps (Δ*HR*_*n*_, Δ*HR*_*n*+1_), where Δ*HR*_*n*_ = *HR*_*n*+1_−*HR*_*n*_. This representation is particularly suited to detecting premature atrial contractions (PAC) and premature ventricular contractions (PVC), which produce a characteristic “kite” trajectory in the plot:

- **SD1 and SD2:** The standard deviations of the point cloud along the minor and major axes of the Poincaré ellipse, capturing short-term and long-term HRV respectively.
- **SD ratio:** The ratio *SD*1/*SD*2, which characterizes the shape of the Poincaré plot.
- **Kite count and kite angular deviation:** The number of kite-pattern triplets (i.e. consecutive quadrant transitions of the form 1 → 2 → 3 or 6 → 4 → 5) and the standard deviation of the trajectory angles at those triplets, encoding the frequency and consistency of ectopic beat patterns.
- **Zero-quadrant ratio:** The proportion of points satisfying |Δ*HR*_*n*_| < 5bpm and |Δ*HR*_*n*+1_| < 5bpm, which reflects the density of the NSR cluster around the origin.

To better illustrate the discriminative power of these features, three segments labeled as NSR, AF, and PAC/PVC were randomly extracted from the database and their Poincaré plots computed under both representations, as shown in Figure 1. The top row displays the classical IPI Poincaré plot (IPI_*n*_ vs. IPI_*n*+1_), on which the SD1 and SD2 axes of the rotated covariance ellipse are overlaid following (Brennan et al., 2001). The bottom row shows the Δ*HR* Poincaré plot of Han et al. (Han et al. 2020), partitioned into the nine regions used to compute the kite-pattern and zero-quadrant features.

**Figure 1:**
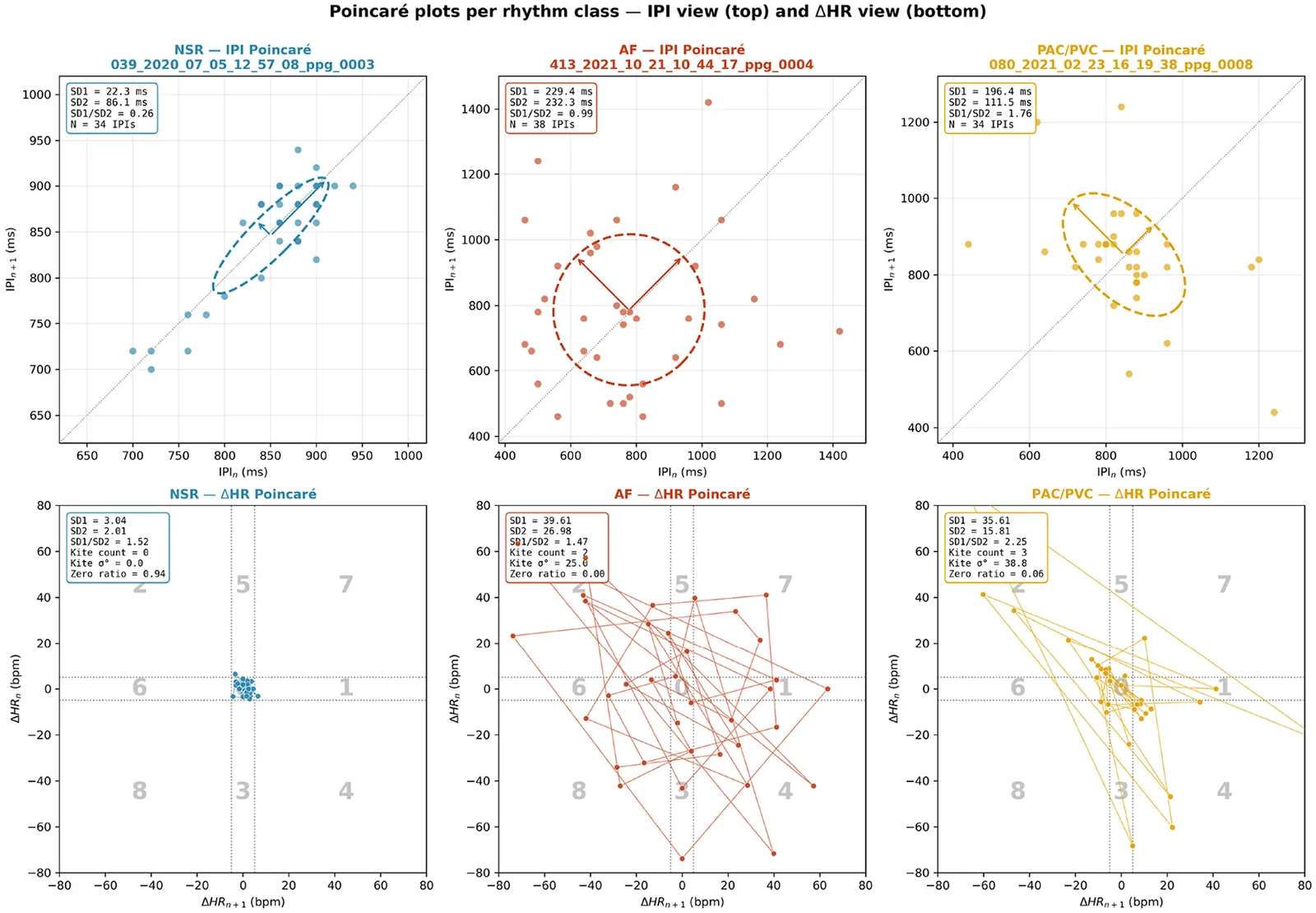
Poincaré plots for one randomly selected segment of each rhythm class (NSR, AF, and PAC/PVC) under two complementary representations.

The three rhythms produce visually distinct signatures consistent with the literature. The NSR segment yields a narrow ellipse aligned with the line of identity (SD1/SD2 = 0.26) and a tightly concentrated Δ*HR* cluster around the origin (zero-quadrant ratio = 0.94, kite count = 0), reflecting the low beat-to-beat variability of sinus rhythm modulated only by respiratory sinus arrhythmia. The AF segment exhibits a wide, near-circular IPI cloud (SD1/SD2 = 0.99) and a Δ*HR* trajectory that spans all eight outer regions with a zero-quadrant ratio of 0.00, characteristic of the stochastic, uncorrelated pulse intervals produced by fibrillatory atrial activity. The PAC/PVC segment shows the expected “kite” geometry: a dense NSR-like cluster centered near the line of identity is accompanied by off-diagonal points corresponding to short premature beats and the ensuing compensatory pauses, yielding SD1/SD2 = 1.76 (i.e., SD1 dominating SD2, the inverse of NSR) and a non-zero kite count of three in the Δ*HR* representation.

#### 4.2.2 Classification

The Random Forest hyperparameters, including the number of trees, maximum tree depth, minimum number of samples required to split an internal node, and minimum number of samples required at a leaf, were selected through grid search within the training fold of each cross-validation direction. The search space comprised *n*_estimators_∈{100, 250, 500}, maximum depth ∈{6, 10, 15, 20, 30, 50}, minimum samples per split ∈{2, 5, 10, 15, 20, 25, 30}, and minimum samples per leaf ∈{2, 5, 10, 15, 20, 25, 30}, restricted to combinations in which the split threshold was at least as large as the leaf threshold, yielding 504 candidate configurations.

For each cross-validation direction, candidate configurations were trained on the training subset of the corresponding fold and selected using the validation subset from the same fold. The opposite fold was never used during hyperparameter selection and was reserved exclusively for subject-independent testing. Model selection was driven by a composite validation score defined as a weighted combination of normalized accuracy, macro F_1_ score, and macro ROC-AUC, with weights of 0.30, 0.40, and 0.30, respectively. The F_1_ score was weighted most heavily to favor configurations that perform well under the pronounced class imbalance between NSR, AF, and PAC/PVC segments.

The retained configuration, 500 trees, a maximum depth of 30, a minimum split size of 10, and a minimum leaf size of 2, was used for the final cross-validation evaluations. Class weights were set to balanced to further compensate for class imbalance during training. It should be noted that this selection procedure targeted predictive quality alone: model size, memory footprint, and inference cost were not part of the composite objective. Consequently, the retained configuration corresponds to the largest ensemble in the search space, 500 trees at a near-maximal depth of 30, which accounts for its comparatively heavy on-disk and memory footprint and leaves clear room for resource-aware optimization.

### 4.3 Multimodal CNN–GRU

We propose a hybrid convolutional–recurrent architecture designed for multi-class arrhythmia classification from multimodal smartwatch signals. The model operates on the multimodal smartwatch representation defined in the General Framework, where each 30-s input segment is represented as a two-channel signal of shape 2 × 1, 500, corresponding to PPG and accelerometer signals sampled at 50 Hz. Data normalization follows the deep learning data handling procedure defined in the General Framework.

The proposed architecture is conceptually similar to the bidirectional GRU-based model introduced by Han et al. (2025), which also employs a convolutional front-end followed by recurrent temporal modeling for multi-class arrhythmia classification. However, several key differences distinguish the present approach. First, the model of Han et al. relies on additional engineered inputs, particularly heart rate (HR) and derived HR representations, alongside the PPG and accelerometer signals, whereas the proposed architecture operates directly on raw PPG and accelerometer signals in a fully end-to-end manner. Second, the proposed model incorporates a temporal attention mechanism, highlighted in orange in Figure 2, to emphasize informative signal regions, which is not present in the baseline architecture. These differences enable the model to learn discriminative features directly from raw smartwatch signals while reducing dependency on handcrafted preprocessing.

**Figure 2:**
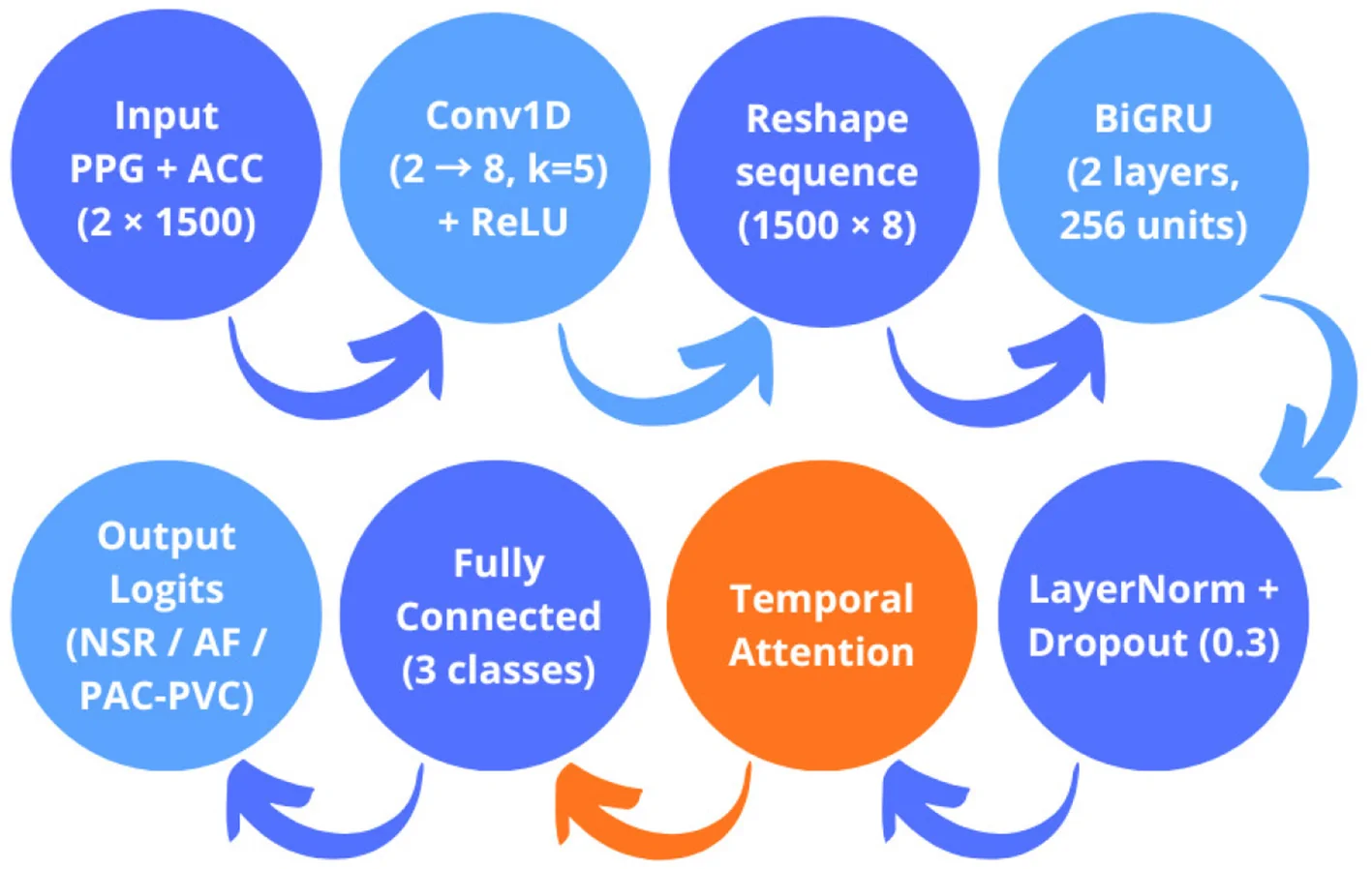
Scheme of the multimodal CNN–GRU approach. The temporal attention module is highlighted in orange to emphasize the architectural component added for adaptive temporal weighting.

The architecture begins with a one-dimensional convolutional layer that maps the two input channels to eight learned feature channels using a kernel size of five, stride one, and zero-padding of two, preserving the temporal length of 1,500 samples. A rectified linear unit activation is applied after convolution. This front-end extracts short-range morphological features from both the PPG and accelerometer streams while enabling early fusion of multimodal information.

The convolutional output, of shape 8 × 1, 500, is rearranged into a temporal-major sequence of 1,500 time steps, each represented by an eight-dimensional feature vector. This sequence is processed by a bidirectional gated recurrent unit (GRU) network with two stacked recurrent layers and 256 hidden units in each direction. Dropout with probability 0.2 is applied between recurrent layers. Bidirectional processing yields a 512-dimensional representation at each time step, allowing the network to model temporal dependencies in both forward and backward directions.

Following the recurrent stage, the time-wise feature vectors are normalized using layer normalization and passed through dropout with probability 0.3. Temporal aggregation is performed using a learnable attention mechanism. Specifically, each 512-dimensional hidden representation is projected to a scalar attention score through a linear layer, and a softmax function is applied across the temporal axis to obtain normalized attention weights. The final sequence representation is computed as the weighted sum of all recurrent outputs, yielding a 512-dimensional embedding that emphasizes the most informative temporal regions for rhythm discrimination.

The resulting sequence-level embedding is passed to a fully connected classification layer, Linear(512 → 3), which produces logits for the three target rhythm categories: normal sinus rhythm, atrial fibrillation, and premature atrial or ventricular contractions.

Training follows the two-fold subject-independent cross-validation protocol defined in the General Framework. The model is trained using the Adam optimizer with a learning rate of 1 × 10^−5^ and cross-entropy loss. The learning rate is adaptively reduced using a ReduceLROnPlateau scheduler with factor 0.5 and patience of two epochs, while early stopping based on validation loss with patience of 10 epochs is employed to prevent overfitting. The implementation uses PyTorch data loaders with two worker processes and prefetching for efficient batch construction.

Overall, the model integrates local feature extraction through convolution, long-range temporal modeling through bidirectional recurrent processing, and adaptive temporal weighting through attention into a unified multimodal architecture for wearable arrhythmia classification. A schematic representation of the proposed architecture is shown in Figure 2, while the detailed configuration required for reproducibility is provided in Table 2.

**Table 2:** Detailed configuration of the proposed CNN–GRU architecture for a single input segment of shape 2 × 1, 500.

Component	Configuration	Input shape	Output shape
Input segment	PPG and ACC channels, 30 s at 50 Hz	–	2 × 1, 500
Convolutional front-end	Conv1D with eight filters, kernel size 5, stride 1, padding 2, and followed by ReLU	2 × 1, 500	8 × 1, 500
Sequence rearrangement	Channel-first to temporal-major representation	8 × 1, 500	1, 500 × 8
Bidirectional recurrent block	BiGRU with two layers, 256 hidden units per direction, and inter-layer dropout *p* = 0.2	1, 500 × 8	1, 500 × 512
Normalization and regularization	Layer normalization over 512 features, followed by dropout *p* = 0.3	1, 500 × 512	1, 500 × 512
Temporal attention	Linear projection 512 → 1, softmax over time, and weighted temporal aggregation	1, 500 × 512	512
Classification layer	Fully connected layer producing logits for NSR, AF, and PAC/PVC, Linear(512 → 3)	512	3

### 4.4 Multimodal CNN–BiLSTM

We propose a second hybrid convolutional–recurrent architecture based on bidirectional long short-term memory (LSTM) modeling and temporal attention. The CNN–BiLSTM model follows the same end-to-end multimodal processing framework as the CNN–GRU architecture, operating directly on the two-channel smartwatch representation defined in the General Framework. Each 30-s input segment has shape 2 × 1, 500, corresponding to PPG and accelerometer signals sampled at 50 Hz. Data normalization follows the same deep learning data handling procedure defined in the General Framework.

The purpose of this architecture is to evaluate whether replacing the GRU block with a bidirectional LSTM block improves temporal modeling in noisy smartwatch recordings. While both recurrent architectures process the same convolutional features and use the same attention-based temporal aggregation strategy, the LSTM block includes explicit input, forget, and output gates, which may support the modeling of longer temporal dependencies and more subtle rhythm irregularities.

The architecture begins with a one-dimensional convolutional layer that maps the two input channels to eight learned feature channels using a kernel size of five, stride one, and padding two, preserving the temporal length of 1,500 samples. A rectified linear unit activation is applied after convolution. As in the CNN–GRU model, this convolutional front-end extracts short-range morphological features from both the PPG waveform and accelerometer signal while enabling early fusion of multimodal information.

The convolutional output, of shape 8 × 1, 500, is rearranged into a temporal-major sequence of 1,500 time steps, each represented by an eight-dimensional feature vector. This sequence is processed by a bidirectional LSTM network with two stacked recurrent layers and 128 hidden units in each direction. Dropout with probability 0.2 is applied between recurrent layers. Bidirectional processing yields a 256-dimensional representation at each time step, allowing the model to capture temporal dependencies in both forward and backward directions.

Following the recurrent stage, the time-wise feature vectors are normalized using layer normalization and passed through dropout with probability 0.3. Temporal aggregation is performed using a learnable attention mechanism. Specifically, each 256-dimensional hidden representation is projected to a scalar attention score through a linear layer, and a softmax function is applied across the temporal axis to obtain normalized attention weights. The final sequence representation is computed as the weighted sum of all recurrent outputs, yielding a 256-dimensional embedding that emphasizes the most informative temporal regions for rhythm discrimination.

The resulting sequence-level embedding is passed to a fully connected classification layer, Linear(256 → 3), which produces logits for the three target rhythm categories: normal sinus rhythm, atrial fibrillation, and premature atrial or ventricular contractions.

Training follows the same two-fold subject-independent cross-validation protocol used for the CNN–GRU model and defined in the General Framework. The model is trained using the Adam optimizer with a learning rate of 1 × 10^−5^ and cross-entropy loss. The learning rate is adaptively reduced using a ReduceLROnPlateau scheduler with factor 0.5 and patience of two epochs, while early stopping based on validation loss with patience of ten epochs is employed to prevent overfitting. The implementation uses PyTorch data loaders with two worker processes and prefetching for efficient batch construction. The learning rate, hidden size, number of recurrent layers, and dropout values reported above were selected empirically through iterative experimentation on the validation split; a more exhaustive hyperparameter search is left for future work.

Overall, the model integrates local feature extraction through convolution, long-range temporal modeling through bidirectional LSTM processing, and adaptive temporal weighting through attention into a unified multimodal architecture for wearable arrhythmia classification. A schematic representation of the proposed architecture is shown in Figure 3, where the bidirectional LSTM block is highlighted in orange to emphasize the main architectural component that differentiates this model from the CNN–GRU variant. The detailed configuration required for reproducibility is provided in Table 3.

**Figure 3:**
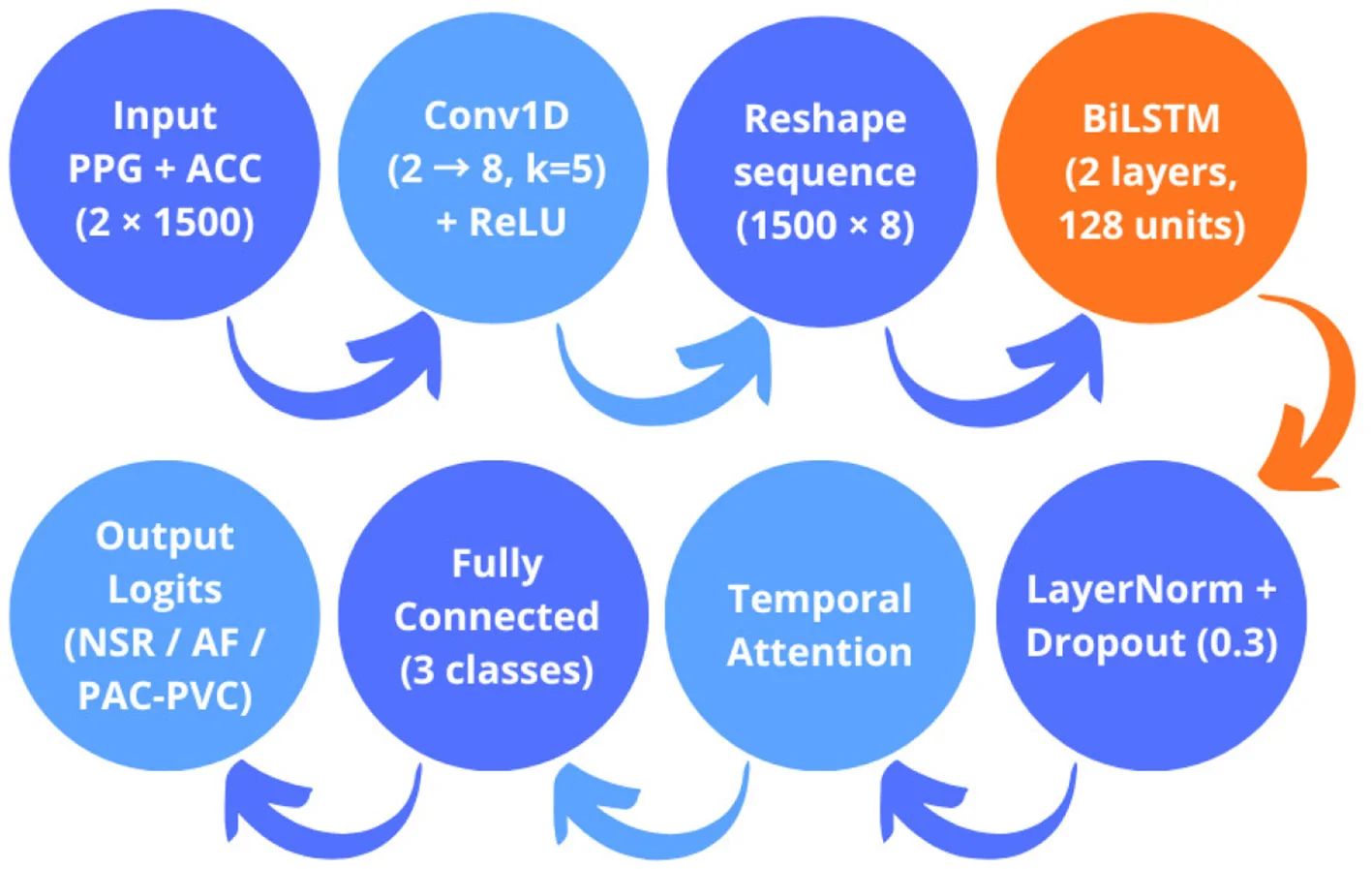
Scheme of the multimodal CNN–BiLSTM approach. The bidirectional LSTM block is highlighted in orange to emphasize the main architectural difference from the CNN–GRU model.

**Table 3:** Detailed configuration of the proposed CNN–BiLSTM architecture for a single input segment of shape 2 × 1, 500.

Component	Configuration	Input shape	Output shape
Input segment	PPG and ACC channels, 30 s at 50 Hz	–	2 × 1, 500
Convolutional front-end	Conv1D with eight filters, kernel size 5, stride 1, padding 2, and followed by ReLU	2 × 1, 500	8 × 1, 500
Sequence rearrangement	Channel-first to temporal-major representation	8 × 1, 500	1, 500 × 8
Bidirectional recurrent block	BiLSTM with two layers, 128 hidden units per direction, and inter-layer dropout *p* = 0.2	1, 500 × 8	1, 500 × 256
Normalization and regularization	Layer normalization over 256 features, followed by dropout *p* = 0.3	1, 500 × 256	1, 500 × 256
Temporal attention	Linear projection 256 → 1, softmax over time, and weighted temporal aggregation	1, 500 × 256	256
Classification layer	Fully connected layer producing logits for NSR, AF, and PAC/PVC, and Linear(256 → 3)	256	3

## 5 Results

Unless otherwise specified, classification results are reported under the two-fold subject-independent cross-validation protocol defined in the General Framework. Main performance values correspond to the average across the two evaluation directions, Fold 1 → Fold 2 and Fold 2 → Fold 1, while direction-wise results are reported separately to characterize fold-dependent variability.

### 5.1 Random Forest classifier

Training and evaluation follow the protocol described in the General Framework, including subject-independent testing on unseen subjects.

#### 5.1.1 Classification performance

Under two-fold subject-independent cross-validation, the Random Forest achieved an average accuracy of 91.22%, a macro precision of 85.89%, a macro recall of 83.21%, a macro F_1_ score of 84.17%, and a macro ROC-AUC of 97.03%. Direction-wise results were highly stable, with an accuracy of 91.08% for Fold 1 → Fold 2 and 91.35% for Fold 2 → Fold 1, indicating consistent cross-subject generalization across the two predefined folds.

The Random Forest shows strong global discrimination and robust overall separability between rhythm classes, as reflected by its high accuracy and macro ROC-AUC. Its class-wise behavior indicates reliable recognition of NSR and AF, while PAC/PVC remains more difficult to detect. This suggests that the feature-based representation captures many rhythm-relevant patterns, but subtle ectopic events remain challenging under noisy smartwatch recording conditions.

The confusion matrices for the two subject-independent cross-validation directions are shown in Figure 4. They illustrate the model's behavior when generalizing from Fold 1 to Fold 2 and in the reverse Fold 2 to Fold 1 direction. In both directions, the model maintains strong recognition of normal sinus rhythm and atrial fibrillation, while premature contractions remain the most challenging rhythm category. From a clinical perspective, this makes the Random Forest highly competitive as an interpretable CPU-based screening model, while also highlighting the importance of reporting class-balanced and class-wise metrics alongside overall accuracy.

**Figure 4:**
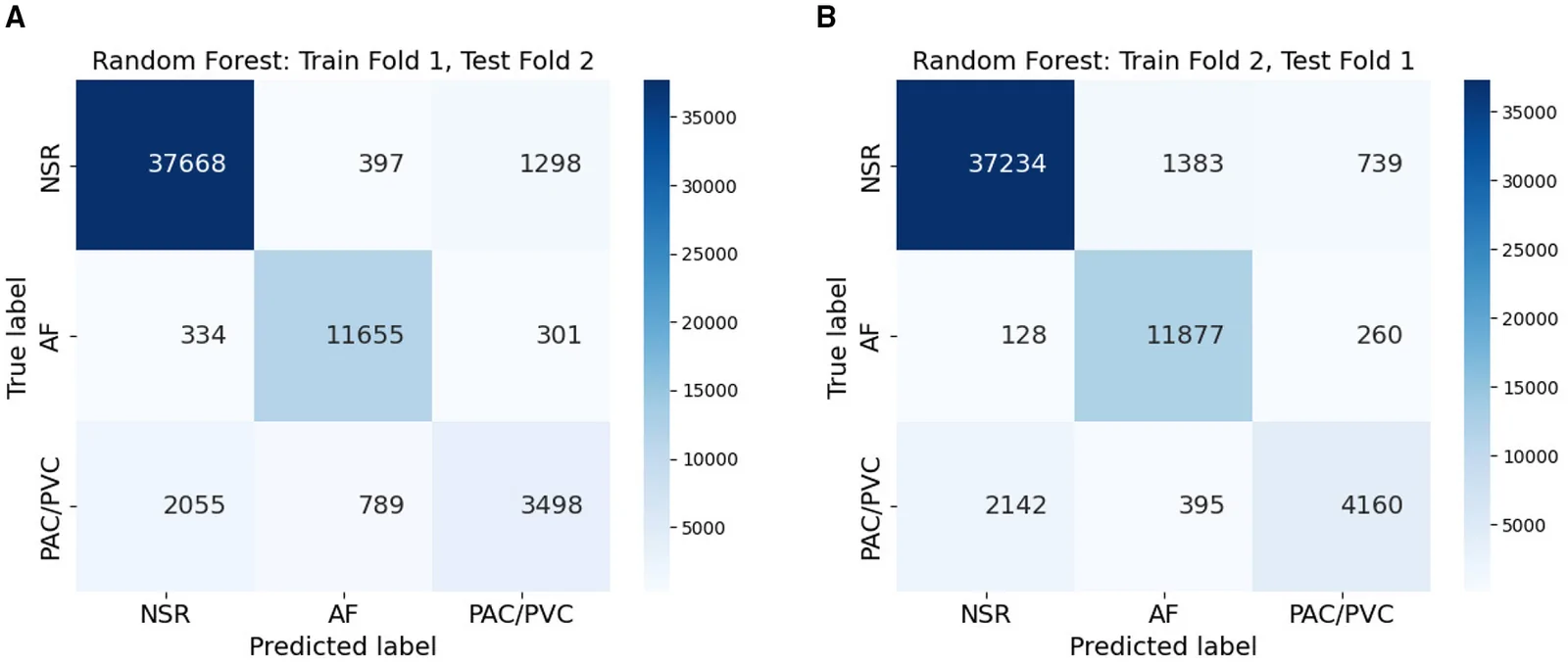
Confusion matrices for the Random Forest model under two-fold subject-independent cross-validation. **(A)** Fold 1 → Fold 2 evaluation, with training and validation performed on Fold 1 and testing on unseen subjects from Fold 2. **(B)** Fold 2 → Fold 1 evaluation, with training and validation performed on Fold 2 and testing on unseen subjects from Fold 1.

#### 5.1.2 Feature importance analysis

The Mean Decrease in Impurity (MDI) importances reveal a clear hierarchy across feature groups. The top two features, poincare_zero_ratio (11.5%) and pnn70 (10.7%), reflect well-established markers of rhythm regularity: NSR heartbeats cluster near the origin of the Poincaré plot (Han et al., 2022), while pNNx statistics are effective indicators of beat-to-beat irregularity (Mietus et al., 2002; Kim et al., 2022). Notably, acc_mag_max ranks third (9.2%), confirming that peak motion intensity is a strong contextual cue for the classifier.

Spectral and entropy-based features (spectral_entropy, markov_ipi, pnn40, markov_pppis, spi, and sampen) occupy the mid-range (4%–7% each), capturing rhythm irregularity and signal complexity. Standard HRV descriptors (nrmssd, poincare_sd1/sd2, cosen, and shen) contribute moderately at 2%–4%, providing corroborating information largely redundant with higher-ranked features.

PPG morphological features—Hjorth parameters, pulse amplitude statistics, and IPI extrema—rank below 2%, suggesting that waveform shape adds limited discriminative power once rhythm-based features are accounted for. The low importance of PAC/PVC-specific Poincaré descriptors (poincare_kite_count, poincare_kite_std_deg) likely reflects class imbalance rather than a true lack of discriminative content.

To further understand the impact of each feature, SHAP analysis was made on a subgroup of 5,000 from Fold-2 for NSR, AF, and PAC/PVC detection separately. The results can be seen in the Figure 5.

**Figure 5:**
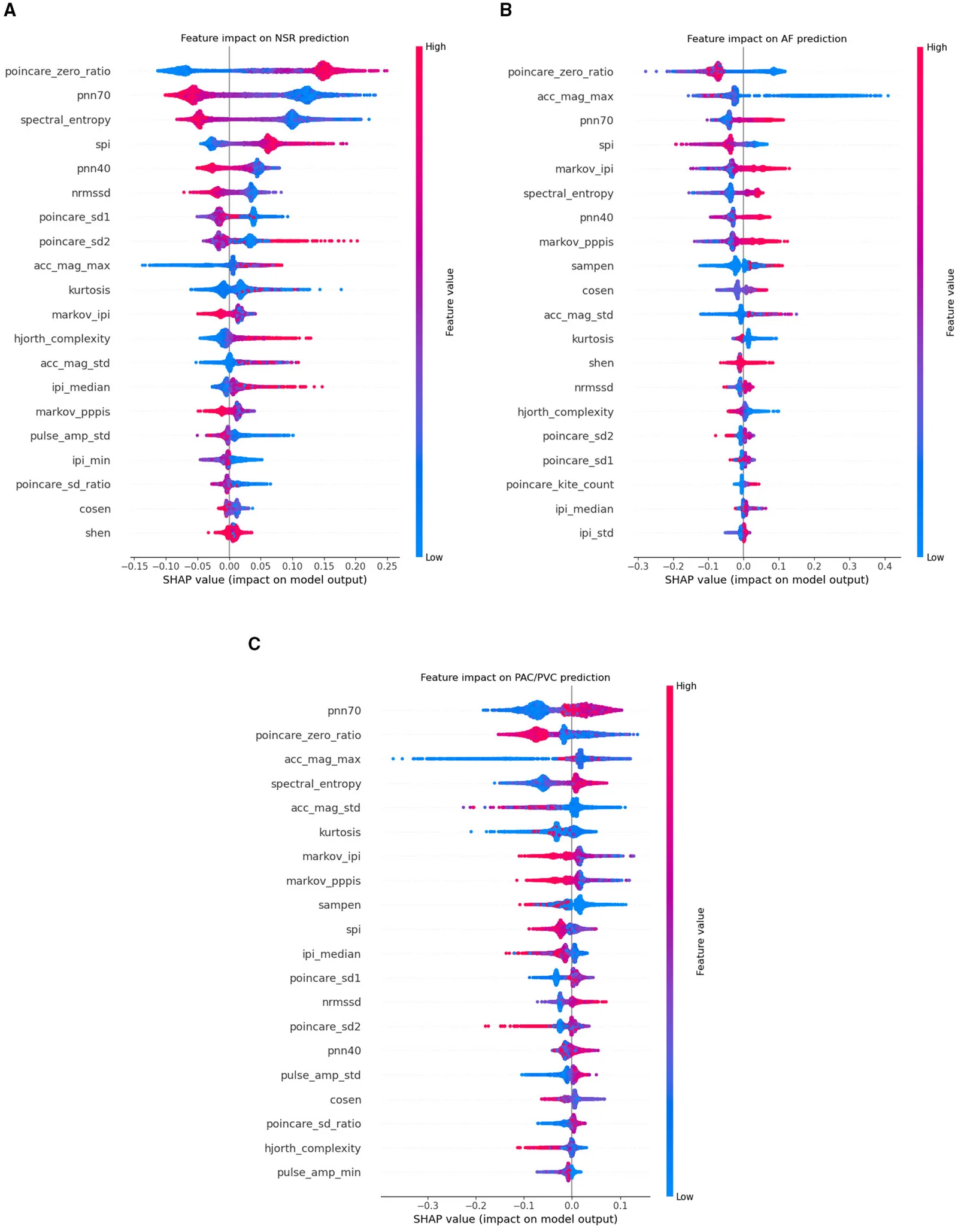
Global feature importance analysis using SHAP summary plots for the Random Forest model. **(A)** Feature impact on normal sinus rhythm (NSR) prediction. **(B)** Feature impact on atrial fibrillation (AF) prediction. **(C)** Feature impact on premature atrial or ventricular contraction (PAC/PVC) prediction.

The SHAP analysis highlights several key findings regarding the model's decision-making process. Foremost, the poincare_zero_ratio emerges as the strongest predictor for Normal Sinus Rhythm (NSR). This aligns perfectly with physiological expectations, as a tightly clustered heart rate represents a regular, healthy rhythm.

Surprisingly, acc_mag_max is identified as the top predictor for Atrial Fibrillation (AF). Because this feature measures physical body motion rather than actual cardiac rhythm, this result must be heavily scrutinized to ensure the model is not relying on confounding noise or movement artifacts to make predictions.

Furthermore, the model evaluates certain features dynamically, as seen with pnn70. This feature exhibits class-dependent directionality, meaning that depending on its exact magnitude, it can drive the prediction toward either NSR or PAC/PVC. This illustrates a highly nuanced and value-specific decision logic within the ensemble.

When evaluating feature categories, spectral_entropy stands out as the most physiologically clean feature derived directly from the PPG waveform. However, as a collective group, the 12 Inter-Pulse Interval (IPI) features carry the most predictive weight, accounting for the highest total SHAP mass across the entire classification model.

#### 5.1.3 Ablation study

While the feature importance analysis describes how the trained model distributes its decisions across the individual descriptors, it does not indicate how much each part of the pipeline contributes to predictive performance. To quantify this, a leave-one-out ablation study was performed over the four feature groups defined in the Feature Extraction section, and additionally over the PPG preprocessing stage, that is the Butterworth band-pass filter followed by the LMS adaptive motion-artifact canceler.

Each variant removes a single feature group, or the preprocessing stage, while keeping everything else unchanged, including the hyperparameters and the random seed, so that every reported difference reflects the marginal contribution of that group given all the others. Removing a feature group reduces the input vector from 29 descriptors to the dimensionality reported in Table 4, whereas the preprocessing variant retains the complete vector but extracts it from the raw PPG signal, so that its difference is attributable to signal conditioning alone. All variants were evaluated on the held-out fold of unseen subjects, with values averaged across both cross-validation directions.

**Table 4:** Leave-one-out ablation of the Random Forest pipeline on the held-out fold of unseen subjects, averaged across both cross-validation directions.

Ablated element	Feature count	Macro F_1_ (%)	AF		PAC/PVC	
			Recall (%)	Precision (%)	Recall (%)	Precision (%)
Reference configuration, absolute values						
None (full pipeline)	29	84.17	95.83	88.87	58.64	74.63
Leave-one-out variants, difference with respect to the full pipeline (percentage points)						
IPI features	17	−2.95	−1.47	−1.72	−6.46	−7.19
PPG waveform features	20	−0.40	−0.41	−0.71	+4.31	−5.27
Poincaré plot features	23	−1.69	−1.02	−0.90	−4.23	−2.21
Accelerometer features	27	**-4.30**	**-5.08**	**-2.13**	−7.24	**-12.83**
PPG preprocessing (raw PPG)	29	−3.82	−2.08	+0.28	**-11.14**	−7.51

The reference row reports absolute values; all other rows report differences with respect to it, so a negative value is the loss incurred by removing that element. The largest degradation per column is shown in bold. NSR is omitted, as its recall varies by at most 1.69 points across variants.

The ablation shows that normal sinus rhythm and atrial fibrillation are largely insensitive to the removal of any individual group, whereas PAC/PVC detection is strongly affected: NSR recall deviates from the full pipeline by at most 1.69 percentage points and AF recall by at most 5.08, while PAC/PVC recall spans more than 15 percentage points. The feature groups and the preprocessing stage therefore contribute mainly to the recognition of the minority class.

The accelerometer group produces the largest overall degradation, despite comprising only two descriptors: its removal decreases AF recall by 5.08 percentage points and PAC/PVC precision by 12.83 percentage points, the largest single drop observed in the study. This corroborates the SHAP analysis, in which acc_mag_max was identified as the strongest contributor to atrial fibrillation prediction, and indicates that the motion context is genuinely exploited by the classifier rather than acting as an incidental correlate of the rhythm labels. Preprocessing contributes comparably, reducing PAC/PVC recall by 11.14 percentage points when the features are extracted from the raw signal; since the feature definitions are identical in this variant, the difference is attributable entirely to signal conditioning. The IPI and Poincaré plot groups follow the same pattern, decreasing PAC/PVC recall by 6.46 and 4.23 percentage points, respectively.

The PPG waveform group is the only one whose removal does not uniformly degrade performance. Excluding it increases PAC/PVC recall by 4.31 percentage points but decreases PAC/PVC precision by 5.27, leaving the PAC/PVC F_1_ score unchanged at 65.64% against 65.67% for the full pipeline. This group therefore trades recall against precision for the ectopic class without yielding a net gain.

These observations should be interpreted with the resolution of the protocol in mind. Each variant was trained once per direction with a fixed seed, and the ablated groups are mutually correlated, since the IPI and Poincaré plot descriptors both estimate beat-to-beat irregularity from the same detected peak series. Differences below approximately one percentage point therefore fall within run-to-run variability, and a small difference indicates redundancy given the remaining groups rather than an absence of discriminative content.

#### 5.1.4 Inference, complexity, and RAM footprint

To assess the deployment suitability of the Random Forest classifier, its structural complexity, inference latency, and memory footprint were profiled under the benchmark protocol defined in the General Framework. The key characteristics are summarized below:

- **Ensemble complexity:** 500 trees with a configured maximum depth of 30 (mean realized tree depth 28.5), comprising 603,837 internal nodes and 604,337 leaves, operating on a 29-dimensional feature vector for three-class classification.
- **On-disk model size:** 107.64 MB (serialized joblib artifact).
- **End-to-end latency (single 30 s segment):** mean 33.08 ms (median 32.99 ms, range 30.92–37.67 ms), decomposed into feature preprocessing at mean 10.36 ms and forest prediction at mean 22.72 ms.
- **Memory footprint (RSS):** loading the model raises the process resident set from a baseline of 309.6 MiB to 592.6 MiB (a model footprint of ≈283 MiB). The peak resident set, the highest *total* RAM the process ever held during the timed run, not an amount added on top of the baseline, reaches 627 MiB, only ≈34 MiB above the post-load value, reflecting transient per-inference working buffers (mean ≈0.29 MB each).

The mean end-to-end time of 33 ms is well within the 30-s segment duration, indicating that the Random Forest pipeline is compatible with near-real-time rhythm screening under the evaluated commodity CPU setup without requiring dedicated accelerators. These measurements provide a deployment-oriented estimate of computational feasibility and motivate further assessment on smartwatch-class or embedded hardware. These figures are compared against the CNN–BiLSTM model in Table 5. Although a direct comparison of computational complexity with the models proposed by Liu et al. (2022), Chen et al. (2024), and the PPG-only approach by Han et al. (2025) is not strictly feasible, an estimated comparison is provided in Table 6.

**Table 5:** Deployment profile of the Random Forest and CNN–BiLSTM pipelines, comparing on-disk size, inference latency, and memory footprint.

Metric	Random Forest	CNN–BiLSTM
Storage		
On-disk model size (MB)	107.64	6.49
**Inference latency, per 30 s segment (ms)**		
Preprocessing (mean)	10.36	0.06
Prediction / forward pass (mean)	22.72	51.81
End-to-end (mean)	33.08	51.87
End-to-end (median)	32.99	51.84
End-to-end (range)	30.92–37.67	51.72–59.39
Memory (resident set size)		
Model footprint (MiB)	283	9.9
Peak RSS (MiB)	627	370.1
Transient per inference (MB)	0.29	0.02

All figures were obtained under the identical single-core, single-thread benchmark defined in the General Framework, using 9,000 timed inferences per profiled model. Latency is reported per 30-s segment; preprocessing and prediction were timed separately and combined in the reported end-to-end latency. The model footprint is the resident set increase after loading, and the peak resident set is the highest total memory the process held during the timed run.

**Table 6:** Model complexity comparison between the proposed models and selected Pulsewatch baselines reported by (Han et al. 2025).

Approach	Number of parameters	GFLOPs
Pulsewatch baselines reported by Han et al. (2025)		
(Liu et al. 2022)	1,626,403	2.36
(Chen et al. 2024)	221,303	19.33
PPG only (Han et al., 2025)	110,696	0.75
Proposed methods		
Random Forest	3,020,685^a^	–^b^
CNN–GRU	1,594,460	4.76^c^
CNN–BiLSTM	538,204	1.60^c^

^a^Non-parametric model; value is a parameter-equivalent computed as 2 × (internal nodes) +*C*×(leaf nodes), *C* = 3 classes, summed across all 500 trees. Not directly comparable to neural network parameter counts.

^b^Omitted: tree-based inference relies on discrete feature comparisons, not floating-point multiply–accumulate operations.

^c^Counted analytically for a single 2 × 1, 500 input segment as twice the number of multiply–accumulate operations in the convolutional front-end, the recurrent layers, the attention projection, and the classifier. The CNN–GRU is the larger of the two proposed networks because its recurrent hidden size is 256 per direction against 128 for the CNN–BiLSTM.

Liu et al. and Chen et al. correspond to previous methods retrained on Pulsewatch by Han et al., while PPG only denotes the most directly comparable Han et al. configuration, operating on photoplethysmography input without additional engineered heart-rate channels. The three proposed methods are reported for the same models and in the same order as in Table 7, so that the effectiveness of each approach can be read against its computational cost. As parameter counts and GFLOPs are not directly comparable between a tree-based ensemble and a neural network, Table 2 can be consulted for a more direct empirical comparison between the Random Forest and CNN–BiLSTM in terms of on-disk size, inference latency, and memory footprint.

### 5.2 Multimodal CNN–GRU

Training and evaluation follow the protocol described in the General Framework, including subject-independent testing on unseen subjects and class balancing through oversampling.

Under two-fold subject-independent cross-validation, the CNN–GRU achieved an average accuracy of 88.93%, a macro precision of 80.48%, a macro recall of 86.85%, a macro F_1_ score of 83.13%, and a macro ROC-AUC of 96.23%, as reported in Table 7. Direction-wise, the model obtained an accuracy of 89.73%, a macro precision of 80.33%, a macro recall of 84.94%, a macro F_1_ score of 82.34%, and a macro ROC-AUC of 96.50% for Fold 1 → Fold 2. In the reverse Fold 2 → Fold 1 direction, it achieved an accuracy of 88.12%, a macro precision of 80.63%, a macro recall of 88.76%, a macro F_1_ score of 83.91%, and a macro ROC-AUC of 95.96%, as shown in Table 8.

**Table 7:** Two-fold subject-independent cross-validation performance comparison between the proposed models and selected Pulsewatch baselines reported by (Han et al. 2025).

Approach	Accuracy (%)	Macro precision (%)	Macro recall (%)	Macro F_1_ (%)	Macro ROC-AUC (%)
Pulsewatch baselines reported by Han et al. Han et al. (2025)					
Liu et al. (2022)	–	84.28	83.19	83.64	–
Chen et al. (2024)	–	78.42	80.51	79.38	–
PPG only (Han et al., (2025)	–	77.40	82.44	79.57	–
Proposed methods					
Random Forest	**91.22**	**85.89**	83.21	**84.17**	**97.03**
CNN–GRU	88.93	80.48	86.85	83.13	96.23
CNN–BiLSTM	87.82	79.88	**87.37**	82.72	96.66

Liu et al. and Chen et al. correspond to previous methods retrained on Pulsewatch by Han et al., while PPG only denotes the most directly comparable Han et al. configuration, operating on photoplethysmography input without additional engineered heart-rate channels. For the proposed methods, values are averaged across Fold 1 → Fold 2 and Fold 2 → Fold 1. For the Han et al. baselines, macro precision, macro recall, and macro F_1_ were computed from the reported class-wise precision and sensitivity values; accuracy and macro ROC-AUC are omitted when not available as model-level metrics. Best available values are shown in bold.

**Table 8:** Direction-wise subject-independent performance of the proposed models.

Approach	Direction	Accuracy (%)	Macro precision (%)	Macro recall (%)	Macro F_1_ (%)	Macro ROC-AUC (%)
Random Forest	Fold 1 → Fold 2	91.08	84.48	81.89	82.92	97.02
Random Forest	Fold 2 → Fold 1	91.35	87.29	84.52	85.42	97.04
CNN–GRU	Fold 1 → Fold 2	89.73	80.33	84.94	82.34	96.50
CNN–GRU	Fold 2 → Fold 1	88.12	80.63	88.76	83.91	95.96
CNN–BiLSTM	Fold 1 → Fold 2	90.08	82.25	87.51	84.34	96.93
CNN–BiLSTM	Fold 2 → Fold 1	85.56	77.51	87.23	81.10	96.38

Fold 1 → Fold 2 denotes training and validation on Fold 1 followed by testing on unseen subjects from Fold 2. Fold 2 → Fold 1 denotes the reverse direction.

The high macro recall obtained by the CNN–GRU indicates that the GRU-based architecture is effective at recovering arrhythmic segments across unseen subjects. In medical decision-support systems, ROC-AUC is particularly relevant because it reflects the intrinsic discriminative capability of the model across all possible classification thresholds. The average macro ROC-AUC of 96.23%, together with direction-wise values above 95.9%, indicates excellent separability between rhythm categories and confirms robust generalization performance under the two-fold subject-independent protocol.

The confusion matrices for the two subject-independent cross-validation directions are presented in Figure 6. The matrices illustrate strong class separation with most predictions concentrated on the diagonal, indicating reliable discrimination between normal sinus rhythm (NSR), atrial fibrillation (AF), and premature atrial or ventricular contractions (PAC/PVC). The error distribution remains relatively balanced without evidence of systematic collapse toward a single class, although residual errors are still concentrated around the distinction between NSR and PAC/PVC.

**Figure 6:**
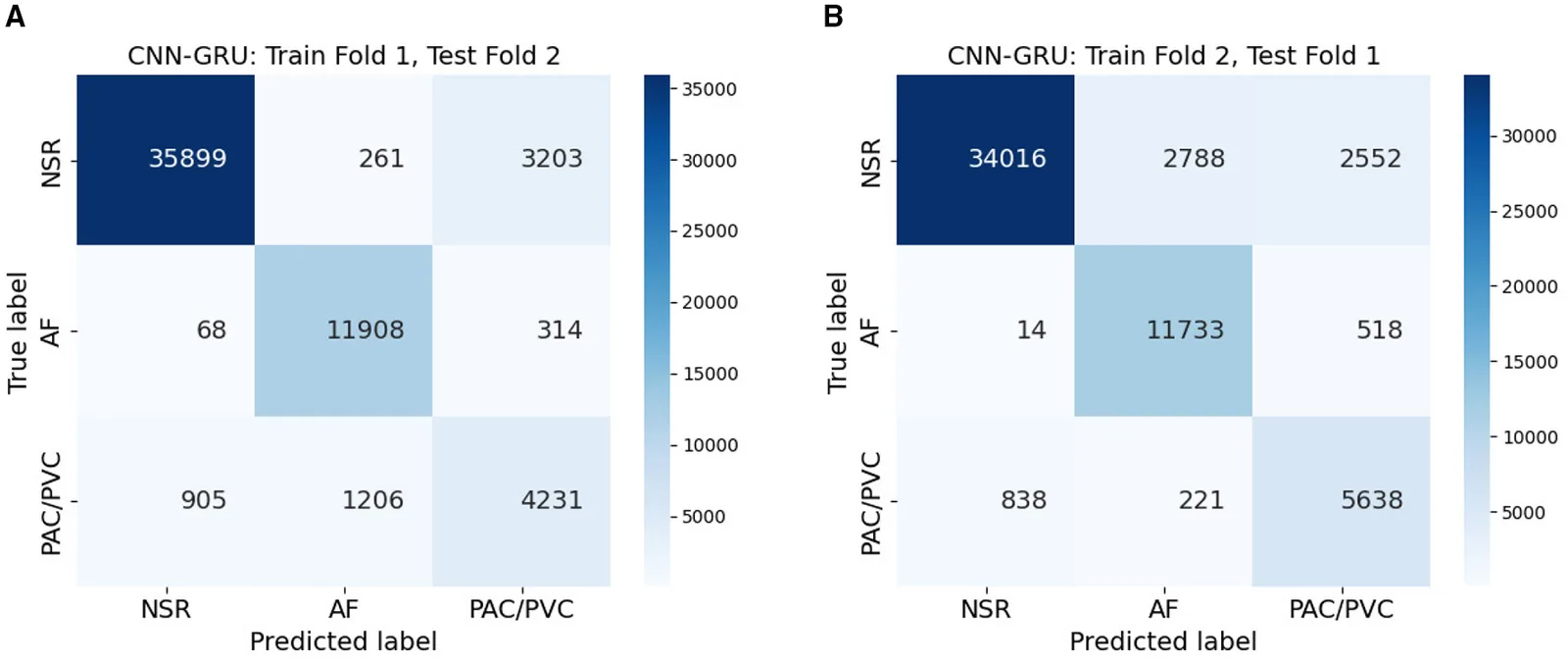
Confusion matrices for the CNN–GRU model under two-fold subject-independent cross-validation. **(A)** Fold 1 → Fold 2 evaluation, with training and validation performed on Fold 1 and testing on unseen subjects from Fold 2. **(B)** Fold 2 → Fold 1 evaluation, with training and validation performed on Fold 2 and testing on unseen subjects from Fold 1.

In the Fold 1 → Fold 2 direction, for normal sinus rhythm, which represents the majority class, 8.14% of samples are misclassified as PAC/PVC and only 0.66% as atrial fibrillation, yielding a total error rate of approximately 8.8%. This indicates that most NSR errors occur toward the ectopic class rather than toward AF.

For atrial fibrillation, which is the most clinically critical class, the model achieves very low misclassification rates in the Fold 1 → Fold 2 direction, with only 0.55% of AF segments incorrectly predicted as NSR and 2.55% as PAC/PVC, resulting in a total error rate of approximately 3.1%. The extremely low proportion of AF-to-NSR errors is particularly important, as it indicates a minimal rate of missed atrial fibrillation events in a screening context.

In contrast, premature atrial and ventricular contractions remain the most challenging class. In the Fold 1 → Fold 2 direction, 14.27% of PAC/PVC samples are misclassified as NSR and 19.02% as AF, corresponding to a total error rate of approximately 33.3%. This reflects the inherent difficulty of distinguishing ectopic beats from both normal rhythm and atrial fibrillation in noisy smartwatch recordings.

Overall, the misclassification pattern shows that the majority of clinically critical errors remain limited, particularly for AF segments misclassified as NSR. This behavior is desirable in rhythm screening systems, as minimizing false negatives for atrial fibrillation is more important than achieving perfect classification of less critical rhythm variations. At the same time, its precision–recall profile suggests that improved sensitivity may come with a higher tendency toward false positive detections, which is consistent with an end-to-end model trained with oversampling. These results support the suitability of the CNN–GRU architecture as a robust temporal modeling baseline for continuous wearable monitoring, while also motivating the evaluation of the CNN–BiLSTM architecture for potentially improved class-balanced sensitivity.

#### 5.2.1 Analysis of misclassified examples

Qualitative examination of representative misclassified segments provides further insight into the nature of residual errors. Certain normal sinus rhythm segments predicted as arrhythmic exhibit irregular morphological characteristics in the PPG waveform despite minimal accelerometer activity, suggesting borderline physiological ambiguity rather than clear algorithmic failure (Figure 7A).

**Figure 7:**
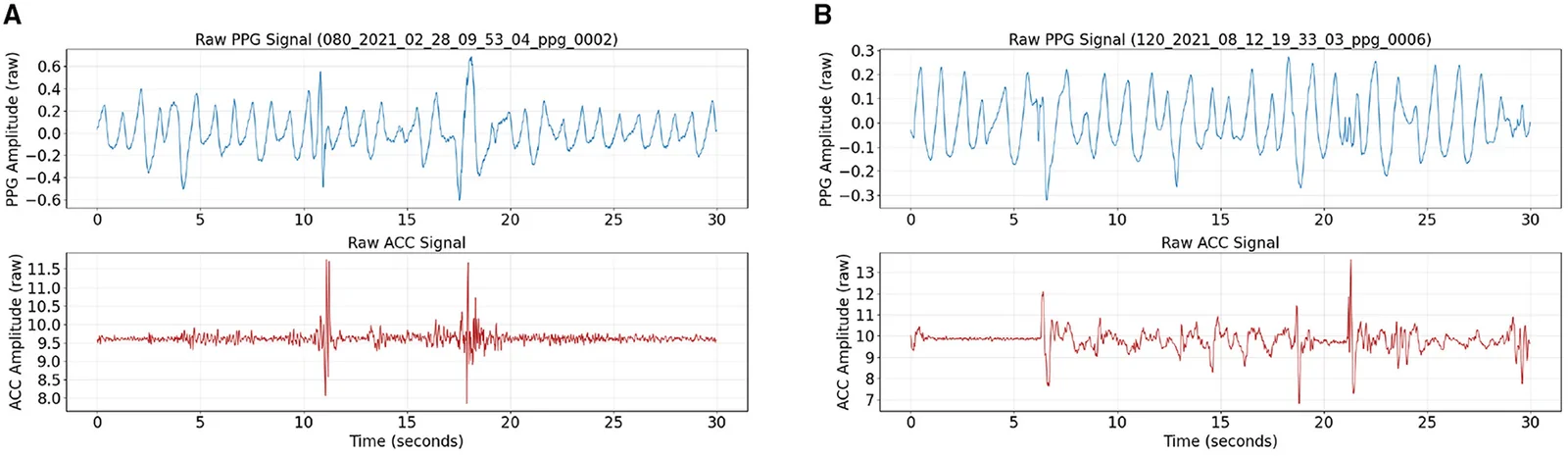
Representative misclassified examples produced by the CNN–GRU model. **(A)** Normal sinus rhythm segment misclassified as arrhythmic (false positive), where the photoplethysmography waveform exhibits mild morphological irregularities despite limited accelerometer activity. **(B)** Arrhythmic segment misclassified as normal rhythm (false negative), where elevated accelerometer activity indicates motion artifacts that distort the photoplethysmography waveform.

This behavior is consistent with known limitations of wrist-based photoplethysmography, where variations in vascular tone, peripheral perfusion, and sensor-skin contact can introduce irregular pulse morphology even in the absence of true arrhythmia (Castaneda et al., 2018; Pereira et al., 2020). In addition, prior studies have shown that PPG-derived pulse intervals may exhibit variability that does not directly correspond to the underlying cardiac rhythm, particularly under real-world ambulatory conditions (Zhang et al., 2019; Lee et al., 2020). As a result, certain segments may present characteristics that resemble arrhythmic patterns despite originating from physiologically normal sinus rhythm.

Similar observations have been reported in wearable arrhythmia detection studies, where borderline or ambiguous segments arise due to the indirect nature of PPG sensing and the presence of subtle waveform distortions that are not captured by accelerometer-based motion indicators (Bashar et al., 2019). These findings support the interpretation that such misclassifications reflect intrinsic signal ambiguity rather than purely model-related errors.

Conversely, some arrhythmic segments misclassified as normal rhythm are associated with substantial motion artifacts, as reflected in elevated accelerometer signals. In these cases, PPG waveform distortion likely masks subtle rhythm-specific features, complicating accurate discrimination (Figure 7B).

Overall, the combination of a high macro ROC-AUC, stable performance under subject-independent evaluation, and balanced confusion matrix distributions indicates that the CNN–GRU architecture provides reliable rhythm discrimination in real-world smartwatch recordings.

However, the residual misclassification patterns, particularly in cases affected by motion artifacts or subtle rhythm irregularities, suggest that further improvements may be obtained through more expressive temporal modeling. While the GRU-based architecture captures short- and mid-range dependencies effectively, these results indicate potential limitations in modeling more complex or long-range temporal dynamics present in wrist-based photoplethysmography signals.

For this reason, we further investigated the CNN–BiLSTM architecture, which provides enhanced temporal representation capacity through its more flexible memory mechanism and bidirectional processing. This allows the model to better capture both past and future context within each segment, potentially improving discrimination between challenging rhythm classes under real-world conditions.

### 5.3 Multimodal CNN–BiLSTM

Training and evaluation follow the protocol described in the General Framework, including subject-independent testing on unseen subjects and class balancing through oversampling.

Under two-fold subject-independent cross-validation, the model achieved an average accuracy of 87.82%, a macro precision of 79.88%, a macro recall of 87.37%, a macro F_1_ score of 82.72%, and a macro ROC-AUC of 96.66%, as reported in Table 7. Direction-wise, the CNN–BiLSTM obtained an accuracy of 90.08%, a macro precision of 82.25%, a macro recall of 87.51%, a macro F_1_ score of 84.34%, and a macro ROC-AUC of 96.93% for Fold 1 → Fold 2. In the reverse Fold 2 → Fold 1 direction, the model achieved an accuracy of 85.56%, a macro precision of 77.51%, a macro recall of 87.23%, a macro F_1_ score of 81.10%, and a macro ROC-AUC of 96.38%, as shown in Table 8.

The CNN–BiLSTM achieved high macro recall, indicating strong sensitivity across rhythm classes. This is particularly relevant in the context of wearable screening, where missed arrhythmic events are clinically more concerning than a moderate increase in false positive detections. At the same time, its precision–recall profile shows that improved sensitivity to minority arrhythmic patterns may be accompanied by additional false positive predictions.

The confusion matrices for the two subject-independent cross-validation directions are presented in Figure 8. The matrices reveal strong separation between rhythm categories, although performance is more fold-dependent than for the Random Forest. This is visible in the decrease in accuracy from 90.08% in the Fold 1 → Fold 2 direction to 85.56% in the Fold 2 → Fold 1 direction, while macro recall remains stable across both directions. This suggests that the model preserves sensitivity to arrhythmic events, even when overall precision and accuracy vary between subject groups.

**Figure 8:**
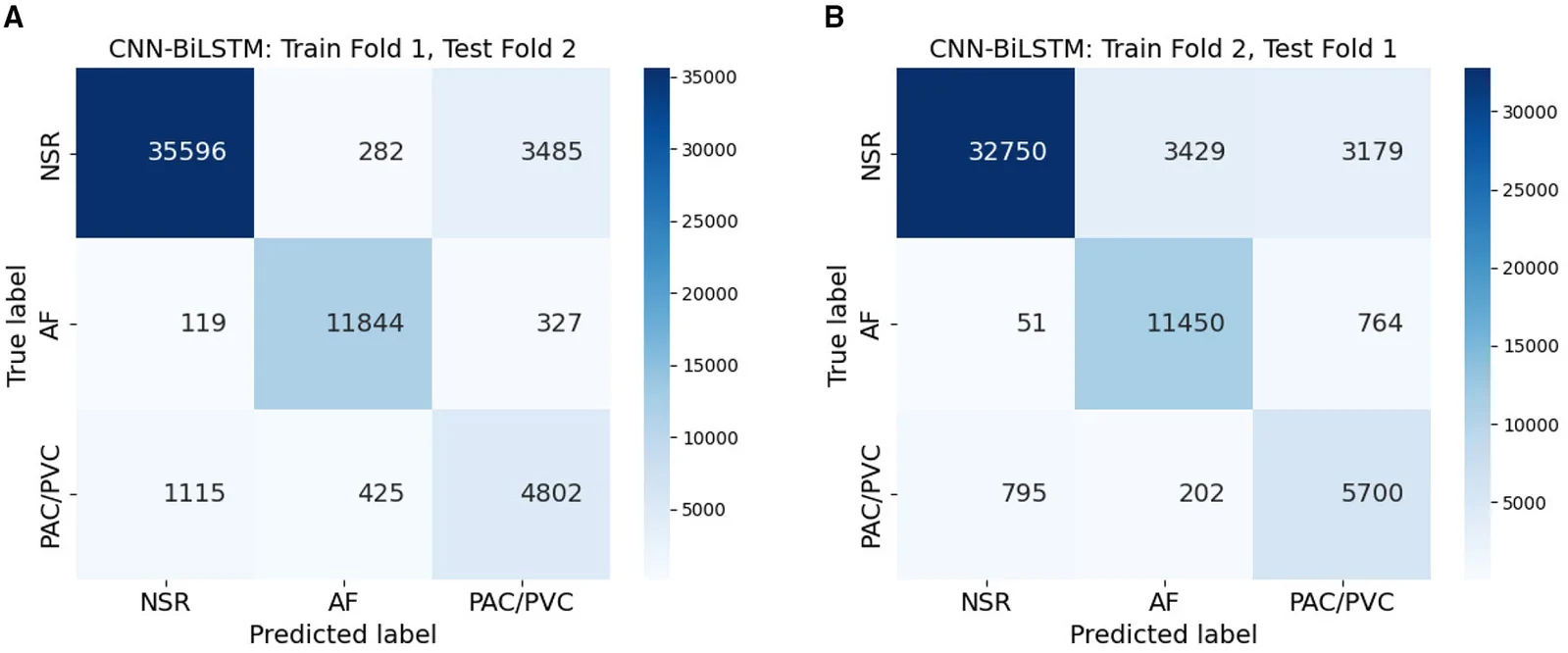
Confusion matrices for the CNN–BiLSTM model under two-fold subject-independent cross-validation. **(A)** Fold 1 → Fold 2 evaluation, with training and validation performed on Fold 1 and testing on unseen subjects from Fold 2. **(B)** Fold 2 → Fold 1 evaluation, with training and validation performed on Fold 2 and testing on unseen subjects from Fold 1.

**Figure 9:**
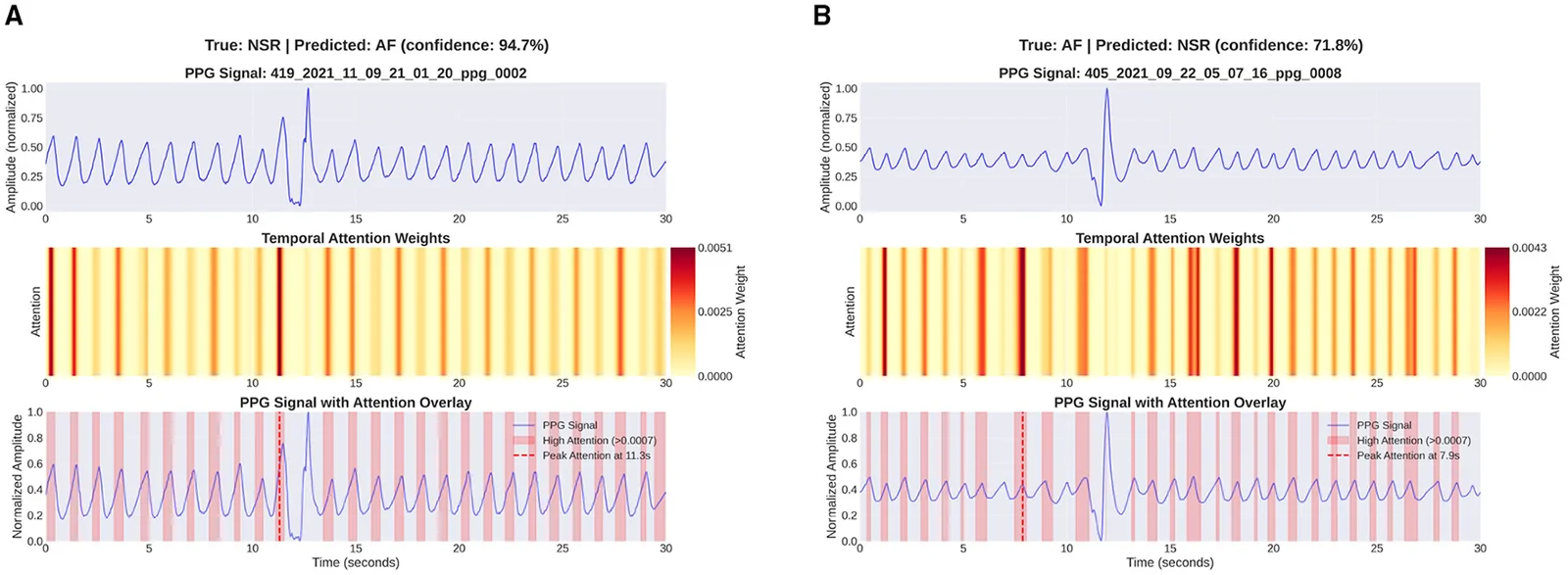
Representative misclassified examples produced by the CNN–BiLSTM model, each showing three horizontally aligned subplots sharing an identical 30-s time axis: the raw normalized PPG signal (top), the temporal attention weight heatmap where color encodes the per-timestep attention weight on a yellow-to-red scale with absolute min/max values indicated on the colorbar (middle), and the PPG signal with the high-attention region shaded in red and the peak-attention instant marked by a dashed red line (bottom). In **(A)**, a normal sinus rhythm segment is misclassified as arrhythmic (false positive), with the attention heatmap concentrating on a localized region consistent with a suspected motion artifact, reflecting spurious feature activation, whereas in **(B)** an atrial fibrillation segment is misclassified as normal rhythm (false negative), with the attention weights remaining diffuse and uniformly low across the entire segment, indicating that the model failed to identify any discriminative temporal features.

In the Fold 1 → Fold 2 direction, the dominant error pathway involves normal sinus rhythm segments misclassified as premature contractions, with 3,485 NSR samples, representing 8.9% of NSR, assigned to the PAC/PVC class. Critically, only 119 atrial fibrillation segments, corresponding to 0.97% of AF samples, were misclassified as normal rhythm. This error type is clinically important because it corresponds to missed atrial fibrillation detections, and its low frequency supports the suitability of the model for continuous cardiac rhythm screening.

The per-class analysis reveals a precision–recall trade-off consistent with the class distribution and the oversampling-based training strategy. In the averaged class-wise evaluation reported in Table 9, the CNN–BiLSTM achieves the highest PAC/PVC recall among all compared models, reaching 79.97%, together with the highest PAC/PVC NPV of 97.34%. This indicates that the model is particularly effective at recovering premature contraction segments and reducing missed ectopic events. However, PAC/PVC precision remains comparatively low at 57.07%, showing that improved sensitivity is obtained at the cost of additional false positive detections.

**Table 9:** Class-wise performance comparison between the proposed models and selected Pulsewatch baselines reported by (Han et al. 2025).

Model	Rhythm	Recall (%)	Specificity (%)	Precision (%)	NPV (%)	Accuracy (%)
Pulsewatch baselines reported by Han et al. (2025)						
Liu et al. (2022)	NSR	**96.03**	91.25	95.83	**91.65**	**94.48**
		AF	90.21	95.98	85.73	97.34	94.76
		PAC/PVC	63.34	96.78	71.27	95.44	93.03
Chen et al. (2024)	NSR	92.88	91.33	95.73	85.97	92.38
		AF	89.21	96.34	86.70	97.09	94.83
		PAC/PVC	59.43	93.30	52.82	94.80	89.50
PPG-only Han et al., (2025)	NSR	90.20	**95.66**	**97.75**	82.33	91.96
		AF	89.65	93.93	79.81	97.14	93.03
		PAC/PVC	67.47	92.93	54.64	95.77	90.08
Proposed methods						
Random Forest	NSR	95.15	87.60	94.14	89.64	92.72
		AF	**95.83**	**96.77**	**88.87**	**98.86**	**96.57**
		PAC/PVC	58.64	**97.48**	**74.63**	94.92	**93.14**
CNN–BiLSTM	NSR	86.96	94.44	97.05	77.94	89.39
		AF	94.89	95.49	85.63	98.58	95.36
		PAC/PVC	**79.97**	92.37	57.07	**97.34**	90.99

Liu et al. and Chen et al. correspond to previous methods retrained and evaluated on Pulsewatch by Han et al., while PPG-only denotes the comparable configuration reported in the same study. For the proposed models, values are averaged across Fold 1 → Fold 2 and Fold 2 → Fold 1. Recall corresponds to class sensitivity in the one-vs.-rest evaluation, NPV denotes negative predictive value, and accuracy denotes class-wise one-vs.-rest accuracy. Best available values for each rhythm and metric are shown in bold.

For atrial fibrillation, the averaged class-wise results show high recall of 94.89% and high NPV of 98.58%, confirming that the model maintains strong sensitivity to the most clinically critical rhythm category. Although the Random Forest achieves the strongest AF performance across the reported class-wise metrics, the CNN–BiLSTM remains highly competitive in AF detection while providing better sensitivity to PAC/PVC segments. This behavior reflects the main advantage of the bidirectional LSTM architecture: its ability to model temporal irregularities that may be subtle, intermittent, or distributed across the 30-s smartwatch segment.

#### 5.3.1 Analysis of misclassified examples

Qualitative examination of representative misclassified segments reveals two dominant failure modes consistent with the characteristics of wrist-based photoplethysmography data.

Certain normal sinus rhythm segments predicted as atrial fibrillation or premature contractions exhibit transient morphological irregularities in the photoplethysmography waveform. Explainability analysis using temporal attention weights and Grad-CAM (Selvaraju et al., 2017) saliency maps indicates that the model focuses on localized signal regions containing artifact-like features, suggesting that transient noise or isolated ectopic beats in otherwise normal recordings trigger false detections (Figure 10A). False positive cases exhibit high saliency variance, with peak Grad-CAM values reaching up to an order of magnitude above the mean, indicating strong but spurious feature activation.

**Figure 10:**
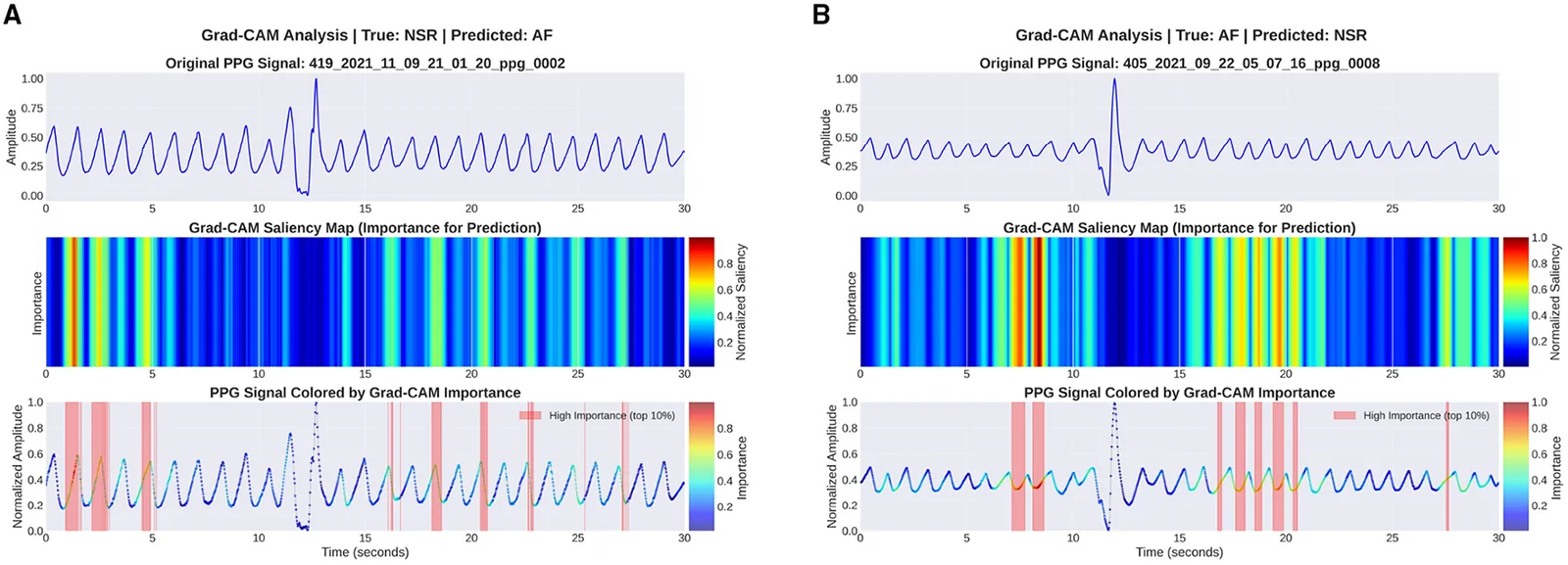
Grad-CAM saliency analysis of the same two misclassified segments shown in Figure 9, each comprising three horizontally aligned subplots sharing an identical 30-s time axis: the raw normalized PPG signal (top), the Grad-CAM saliency map where color encodes the min–max normalized gradient magnitude on a jet scale (middle), and the PPG signal with each sample colored by its saliency and the top-decile regions shaded in red (bottom). Saliency is computed as the channel-averaged absolute gradient of the predicted-class logit with respect to the input, evaluated with dropout disabled so that the maps are deterministic. In **(A)**, the false positive segment (NSR predicted as AF) shows saliency concentrated in the first 5 s, away from the suspected motion artifact at approximately 12 s, with a peak-to-mean saliency ratio of 3.96. In **(B)**, the false negative segment (AF predicted as NSR) concentrates saliency in a band between roughly 15 and 20 s, with a peak-to-mean ratio of 3.34 but an absolute mean saliency approximately twice that of the false positive case.

Conversely, subtle or paroxysmal atrial fibrillation segments misclassified as normal rhythm are characterized by lower and more uniformly distributed saliency, indicating that the model failed to identify discriminative temporal features. These cases likely correspond to where the characteristic irregularity in pulse timing is attenuated or masked by motion artifacts (Figure 10B).

Overall, the combination of a high macro ROC-AUC, maintained atrial fibrillation sensitivity, and interpretable error patterns confirms that the CNN–BiLSTM architecture provides reliable multi-class rhythm discrimination in real-world smartwatch recordings while offering actionable insight into failure modes through built-in explainability mechanisms.

#### 5.3.2 Inference, complexity, and RAM footprint

To assess the deployment suitability of the proposed CNN–BiLSTM architecture, its computational cost, inference latency, and memory footprint were profiled under the same benchmark protocol defined in the General Framework. The key characteristics are summarized below:

- **Trainable parameters:** 538,204.
- **Model size (deployed weights):** 6.49 MB (serialized inference checkpoint).
- **FLOPs per segment:** ≈1.60 GFLOPs (estimated analytically for a single 2 × 1, 500 input; dominated by the bidirectional LSTM layers). This is approximately 2.1 × the computational load of the PPG-only BiGRU reported by Han et al. (2025) (≈0.75 GFLOPs).
- **End-to-end latency (single 30 s segment):** mean 51.87 ms (median 51.84 ms, range 51.72–59.39 ms), with the network forward pass (mean 51.81 ms) dominating and input preprocessing being negligible (≈0.06 ms).
- **Memory footprint (RSS):** loading the model raises the process resident set from a baseline of 309.8 MiB to 319.7 MiB (a model footprint of ≈9.9 MiB). The peak resident set, the highest *total* RAM the process ever held during the timed run, not an amount added on top of the baseline, reaches 370.1 MiB, with a transient per-inference working allocation of only ≈0.02 MB.

The mean end-to-end time of 52 ms is well within the 30-s segment duration, indicating that the model is compatible with near-real-time rhythm screening under the evaluated commodity CPU setup without requiring dedicated accelerators. These measurements provide a deployment-oriented estimate of computational feasibility and motivate further assessment on smartwatch-class or embedded hardware. A direct comparison with the Random Forest deployment profile is provided in Table 2. For a direct comparison of parameters and GFLOPs per segment against the models proposed by Liu et al. (2022), Chen et al. (2024), and the PPG-only approach by Han et al. (2025), refer to Table 6.

### 5.4 Comparative analysis of the investigated approaches

Table 7 summarizes the average two-fold subject-independent performance of the proposed models together with selected Pulsewatch baselines reported by Han et al. (2025). The Liu et al. (2022) and Chen et al. (2024) entries correspond to previous methods retrained and evaluated on Pulsewatch by Han et al., while the PPG-only entry corresponds to the comparable Han et al. configuration operating on photoplethysmography input without additional engineered heart-rate channels. For these baselines, only macro precision, macro recall, and macro F_1_ are included, because a single model-level accuracy and macro ROC-AUC are not available from the reported class-wise results.

From a global performance perspective, the Random Forest achieved the strongest average results among the proposed methods, with the highest accuracy (91.22%), macro precision (85.89%), macro F_1_ score (84.17%), and macro ROC-AUC (97.03%). Its direction-wise results were also highly stable, with accuracies of 91.08% and 91.35% in the two evaluation directions. This indicates that the feature-based pipeline provides robust cross-subject generalization under the Pulsewatch two-fold subject-independent protocol. The strong performance of this model suggests that carefully designed rhythm-variability, morphology, accelerometer, and Poincaré-based descriptors remain highly informative for smartwatch-based arrhythmia detection.

Compared with the Pulsewatch baselines reported by Han et al., the Random Forest achieved the highest macro precision and macro F_1_ among the available values. Its macro F_1_ score of 84.17% slightly exceeded the retrained Liu et al. baseline (83.64%) and was higher than both Chen et al. (79.38%) and the PPG-only Han et al. configuration (79.57%). This improvement is particularly relevant because macro F_1_ balances precision and recall across classes and is therefore more informative than overall accuracy in an imbalanced multi-class setting.

The CNN–GRU and CNN–BiLSTM models showed a different performance profile. Both end-to-end neural architectures achieved higher macro recall than the Pulsewatch baselines reported by Han et al., with average macro recall values of 86.85% for CNN–GRU and 87.37% for CNN–BiLSTM, compared with 83.19% for Liu et al., 80.51% for Chen et al., and 82.44% for the PPG-only Han et al. configuration. The CNN–BiLSTM obtained the highest macro recall among all methods in Table 7, indicating the strongest average sensitivity across rhythm classes. However, its lower macro precision and macro F_1_ score compared with the Random Forest show that this increased sensitivity is obtained at the cost of additional false positive predictions.

The class-wise analysis in Table 9 provides a more detailed view of these trade-offs. For normal sinus rhythm, the strongest recall, negative predictive value, and one-vs.-rest accuracy are obtained by Liu et al., while the PPG-only Han et al. configuration achieves the highest specificity and precision. This shows that the previously reported Pulsewatch baselines remain strong for the majority rhythm class. In contrast, the proposed models are more competitive for clinically relevant arrhythmic classes, particularly atrial fibrillation and PAC/PVC.

For atrial fibrillation, which represents the most clinically critical rhythm in wearable screening, the proposed Random Forest achieved the best values across all reported class-wise metrics: recall (95.83%), specificity (96.77%), precision (88.87%), negative predictive value (98.86%), and one-vs.-rest accuracy (96.57%). This result is clinically important because missed atrial fibrillation episodes may delay diagnostic confirmation and treatment, while excessive false positives may increase unnecessary alerts and clinical workload. The Random Forest therefore provides a strong AF detection profile, combining high sensitivity with high precision.

For PAC/PVC detection, the proposed methods show complementary strengths. The CNN–BiLSTM achieved the highest recall (79.97%) and negative predictive value (97.34%), indicating the strongest ability to recover ectopic segments and reduce missed PAC/PVC cases. In contrast, the Random Forest achieved the highest specificity (97.48%), precision (74.63%), and one-vs.-rest accuracy (93.14%) for PAC/PVC, indicating fewer false positive detections for this class. This distinction highlights a clinically meaningful trade-off: the CNN–BiLSTM is more sensitive to minority ectopic events, whereas the Random Forest is more conservative and precise.

These findings show that no single model dominates all evaluation criteria. The Random Forest provides the strongest overall average performance, the highest macro F_1_ score, and the best atrial fibrillation class-wise profile. The CNN–BiLSTM provides the highest macro recall and the strongest PAC/PVC sensitivity, making it attractive when reducing missed minority arrhythmic events is prioritized. The CNN–GRU remains a competitive end-to-end alternative, particularly in terms of macro recall and ROC-AUC, although it does not surpass the Random Forest in macro precision, macro F_1_, or overall accuracy.

From an application perspective, the preferred model depends on the intended screening objective. If the goal is a stable, interpretable, CPU-oriented model with strong overall performance and high precision, the Random Forest is the most suitable candidate among the investigated approaches. If the priority is increased sensitivity to arrhythmic events, especially PAC/PVC segments, the CNN–BiLSTM offers a clinically relevant alternative, although its lower precision should be considered when designing alerting workflows. Overall, the comparative analysis demonstrates that feature-based and end-to-end temporal models provide complementary strengths for wearable arrhythmia detection, and that both macro-level and class-wise metrics are necessary to characterize their practical value under subject-independent evaluation.

## 6 Discussion

This study provides a systematic comparison between classical machine learning and end-to-end deep learning approaches for multi-class arrhythmia detection from smartwatch photoplethysmography and accelerometer signals under identical evaluation conditions. Unlike many prior studies that focus on binary atrial fibrillation vs. normal sinus rhythm classification, the present work addresses a more challenging multi-class setting involving normal sinus rhythm, atrial fibrillation, and premature atrial or ventricular contractions in noisy, real-world smartwatch recordings. By evaluating all models under a two-fold subject-independent cross-validation protocol, the analysis offers insight into predictive performance and cross-subject generalization across both fold directions, which is critical for real-world deployment.

Among the models developed in this work, the Random Forest classifier achieved the strongest average performance under two-fold subject-independent cross-validation, with the highest accuracy (91.22%), macro precision (85.89%), macro F_1_ score (84.17%), and macro ROC-AUC (97.03%) among the proposed methods. This result highlights the strong discriminative power of carefully engineered heart rate variability, morphological, accelerometer-based, and Poincaré-derived features, particularly in the presence of class imbalance where the dominant normal sinus rhythm class contributes significantly to global accuracy. In addition, the Random Forest benefits from high interpretability through SHAP-based analysis, allowing direct inspection of feature contributions. However, despite its strong global performance, the model exhibits lower sensitivity to PAC/PVC segments than the neural architectures, indicating that subtle ectopic patterns remain more difficult to capture using the handcrafted feature representation alone.

The strong global performance of the Random Forest model should also be interpreted in light of its feature-based pipeline. Unlike the deep learning models, the Random Forest does not operate directly on minimally processed waveforms, but benefits from explicit preprocessing, peak detection, motion artifact reduction, and handcrafted descriptors that encode substantial domain knowledge. This was an intentional design choice, since the aim was to compare a well-defined classical wearable-signal pipeline with end-to-end neural models, rather than to evaluate all classifiers on an identical feature representation. Consequently, the results should be understood as a comparison between modeling paradigms: an interpretable feature-engineering approach and raw-waveform deep learning approaches. This distinction is important when interpreting the trade-off between accuracy, interpretability, class-balanced performance, and practical adaptability.

In contrast, the proposed deep learning architectures demonstrate a different class-balanced performance profile, with stronger sensitivity to arrhythmic classes. The CNN–BiLSTM model achieved the highest macro recall among the proposed methods, reaching 87.37%, while the CNN–GRU remained competitive with a macro recall of 86.85%. These results indicate that end-to-end convolutional–recurrent models are effective at recovering arrhythmic segments from raw smartwatch waveforms. However, both neural architectures obtained lower macro precision than the Random Forest, suggesting a higher tendency toward false positive detections. Notably, the CNN–GRU and CNN–BiLSTM operate directly on raw multimodal smartwatch signals without relying on engineered heart-rate representations, making them suitable for scalable rhythm monitoring pipelines where explicit feature engineering is reduced.

Class imbalance was addressed using random oversampling because it provides a simple and transparent way to increase the exposure of the end-to-end models to minority rhythm classes during training, while leaving the validation and test distributions unchanged. This choice was also appropriate for raw smartwatch time series, where synthetic interpolation methods such as SMOTE may generate waveform patterns that are difficult to interpret physiologically. The high macro recall obtained by both CNN–GRU and CNN–BiLSTM, together with the strongest PAC/PVC recall achieved by CNN–BiLSTM in the class-wise analysis, suggests that oversampling contributed to better minority-class recognition. Nevertheless, the lower precision observed for PAC/PVC also indicates that alternative imbalance-aware training strategies require further investigation.

A direct comparison with the Pulsewatch baselines reported by Han et al. (2025) provides additional insight into the advantages of the proposed architectures. Since the published comparative table reports class-wise quantities for the baseline methods, macro precision, macro recall, and macro F_1_ could be computed, whereas a single model-level accuracy and macro ROC-AUC could not be reconstructed. Under these available metrics, the Random Forest achieved the highest macro precision (85.89%) and macro F_1_ score (84.17%), slightly exceeding the retrained Liu et al. baseline in macro F_1_ (83.64%) and outperforming the Chen et al. and PPG-only Han et al. configurations. The CNN–BiLSTM achieved the highest macro recall (87.37%), indicating stronger average sensitivity across rhythm classes.

The class-wise analysis further emphasizes the clinical relevance of these differences. For atrial fibrillation detection, the Random Forest achieves the strongest profile among the compared methods, with a recall of 95.83%, specificity of 96.77%, precision of 88.87%, NPV of 98.86%, and one-vs.-rest accuracy of 96.57%. This indicates that the feature-based model is highly effective for AF detection, combining high sensitivity with comparatively high precision. For PAC/PVC detection, the CNN–BiLSTM achieves the highest recall (79.97%) and NPV (97.34%), indicating a better ability to recover subtle ectopic patterns. However, its PAC/PVC precision remains lower (57.07%) than that of the Random Forest (74.63%), showing that this gain in sensitivity is obtained at the cost of additional false positive detections.

Beyond aggregate classification metrics, the clinical relevance of the models depends on the direction and consequences of misclassification errors. In wearable screening, the most critical pathway is the classification of atrial fibrillation as normal sinus rhythm, since this corresponds to a missed abnormal rhythm and may delay further diagnostic evaluation. This is particularly important because AF may be intermittent, paroxysmal, or asymptomatic, and short clinical recordings may fail to capture relevant episodes (Hindricks et al., 2021; Perez et al., 2019; Lubitz et al., 2022). The averaged class-wise results show that both the Random Forest and CNN–BiLSTM maintain high AF recall, while the CNN–GRU also provides strong sensitivity under the two-fold subject-independent protocol. For PAC/PVC, the CNN–BiLSTM reduces the risk of missed ectopic events, whereas the Random Forest reduces false positive PAC/PVC detections through higher precision and specificity. These complementary error profiles show that the preferred model depends on the intended screening objective.

The consequences of non-critical or less critical error pathways are different. AF segments classified as PAC/PVC remain rhythm-specific errors, but they are still detected as abnormal and may therefore trigger additional review. Conversely, false positive predictions, such as NSR segments classified as AF or PAC/PVC, may increase unnecessary alerts, user anxiety, and clinician workload, but they are generally less harmful than missed AF episodes in a screening context. These error patterns should also be interpreted in relation to the limitations of wrist-based photoplethysmography, which is an indirect and motion-sensitive measurement. Variations in vascular tone, peripheral perfusion, sensor–skin contact, and motion artifacts can introduce irregular pulse morphology even in the absence of true arrhythmia (Castaneda et al., 2018; Pereira et al., 2020; Zhang et al., 2019; Lee et al., 2020).

From a modeling perspective, these results suggest that feature-based and end-to-end temporal learning strategies capture complementary aspects of smartwatch rhythm data. The Random Forest benefits from explicit preprocessing and domain-specific descriptors, which provide strong global performance and interpretability. The CNN–GRU and CNN–BiLSTM models, in turn, learn temporal representations directly from raw two-channel waveforms, reducing the need for handcrafted intermediate features. The bidirectional LSTM component, combined with convolutional feature extraction and temporal attention, enables the model to capture both local morphological patterns and longer temporal dependencies, which may explain its improved sensitivity to PAC/PVC events. Furthermore, the use of attention mechanisms and Grad-CAM saliency maps provides an additional level of interpretability, allowing visualization of the temporal regions that contribute most strongly to classification decisions. More broadly, the comparison highlights how architectural design choices influence cross-subject generalization, robustness to motion artifacts, and explainability, emphasizing the complementary strengths of classical and deep learning approaches for wearable arrhythmia detection.

The deployment analysis further supports the practical relevance of these findings, while also defining the hardware context in which the reported runtimes should be interpreted. Both models were profiled under the identical single-core, single-thread benchmark defined in the General Framework, with preprocessing and prediction timed jointly for each 30-s segment, so their runtimes are directly comparable under the evaluated CPU conditions. The Random Forest pipeline achieved a mean end-to-end time of 33.08 ms, including preprocessing, feature extraction, and classification, indicating a computationally efficient and interpretable screening pipeline under this benchmark. The CNN–BiLSTM model, although more computationally demanding at approximately 1.60 GFLOPs per segment, achieved a mean end-to-end latency of 51.87 ms. As summarized in Table 6, this floating-point cost remains below that of the Pulsewatch baselines retrained by Han et al. (2025), namely Liu et al. (2022) at 2.36 GFLOPs and Chen et al. (2024) at 19.33 GFLOPs, while exceeding their more compact PPG-only configuration at 0.75 GFLOPs. Since both inference times are far below the 30-second segment duration, the results suggest that both approaches are compatible with near-real-time rhythm screening under the evaluated conditions.

As summarized in Table 2, the comparison reveals a clear resource trade-off: the Random Forest is faster in latency but substantially heavier in storage and memory, with a 107.64 MB model and a 627 MiB peak resident set, whereas the CNN–BiLSTM is slower but markedly more compact, with a 6.49 MB model and a 370 MiB peak resident set. These results are encouraging from a deployment perspective, but they should be interpreted as a controlled CPU-based feasibility assessment rather than as a definitive validation of on-watch execution. Actual smartwatch or embedded deployment may be affected by platform-specific constraints, including processor architecture, available memory, energy consumption, thermal behavior, operating-system scheduling, and sustained background execution. Therefore, while the reported results support the feasibility of near-real-time processing at the pipeline level, dedicated evaluation on smartwatch-class or embedded hardware remains necessary before drawing conclusions about fully on-device deployment.

Overall, the findings highlight a clear trade-off between accuracy, interpretability, clinical error profile, and practical usefulness. The Random Forest achieves the strongest overall average performance and remains attractive for interpretable CPU-based screening, with particularly strong AF detection. In contrast, the CNN–BiLSTM offers increased sensitivity to minority arrhythmic events, especially PAC/PVC segments, while maintaining a substantially smaller storage and memory footprint. The CNN–GRU remains a competitive end-to-end alternative, particularly in terms of macro recall and ROC-AUC. These results support the feasibility of artificial intelligence–driven cardiac rhythm screening systems capable of operating on smartwatch-acquired signals in free-living conditions.

Several limitations should be acknowledged. All experiments were conducted on the Pulsewatch dataset using a two-fold subject-independent cross-validation protocol designed to assess generalization to unseen individuals across the two predefined folds. Although this provides a stronger evaluation than a single train-test direction and avoids subject overlap between training and testing, external validation on independent datasets remains necessary to determine robustness across different populations, devices, acquisition protocols, signal-quality distributions, and annotation procedures. In addition, comparison with prior Pulsewatch baselines is limited by the metrics available in the published comparative table of Han et al., therefore, some model-level quantities, such as overall accuracy and macro ROC-AUC, could not be reconstructed for the reported baselines. Finally, premature atrial and ventricular contractions remain more difficult to classify than NSR and AF, indicating the need for improved modeling of subtle and heterogeneous ectopic patterns. The deployment benchmark was conducted on general-purpose CPU hardware under controlled single-core, single-thread conditions. Although this protocol enables a fair comparison between the evaluated pipelines, actual smartwatch or embedded deployment may be influenced by platform-specific constraints such as processor architecture, available memory, energy consumption, thermal behavior, operating-system scheduling, and sustained background execution. The hyperparameter selection procedure should also be considered when interpreting the results. The Random Forest configuration was selected through grid search using a composite predictive criterion based on accuracy, macro F_1_ score, and macro ROC-AUC, but resource-related objectives such as storage size, memory footprint, and inference latency were not included. For the deep learning models, the learning rate, recurrent hidden size, number of recurrent layers, and dropout values were selected empirically based on validation behavior and computational feasibility, rather than through an exhaustive hyperparameter search. Moreover, because the Random Forest and deep learning models represent different modeling paradigms, they do not use equivalent preprocessing strategies. The comparison should therefore be interpreted as an evaluation of complete modeling pipelines, rather than as an isolated comparison of classifiers under identical input representations.

Future work will focus on extending evaluation to additional datasets, including cross-dataset generalization experiments, improving robustness to motion artifacts, and validating the proposed pipelines on embedded or smartwatch-class hardware under realistic memory, energy, thermal, and sustained-inference constraints. In particular, future analyses should quantify model robustness under different motion-intensity levels by stratifying test segments into low-, medium-, and high-motion conditions based on accelerometer-derived activity measures. This should be complemented by designing lightweight architectures suitable for real-time inference on wearable devices, including resource-aware hyperparameter selection for the Random Forest to reduce its footprint. Additional ablation experiments should also be performed to isolate the contribution of individual architectural components, including the convolutional front-end, recurrent layers, bidirectional processing, and temporal attention mechanism. Such experiments would clarify the specific role of each component in class-balanced arrhythmia detection and further justify the selected CNN–GRU and CNN–BiLSTM designs. Further analyses should also evaluate classical and neural models under more closely matched preprocessing conditions, in order to better separate the effect of representation learning from the effect of domain-specific feature engineering. Future experiments should also investigate more advanced imbalance-aware training strategies, including focal loss, weighted cross-entropy, class-balanced loss, and physiologically plausible augmentation for raw smartwatch waveforms. In addition, systematic hyperparameter optimization using grid search, random search, or Bayesian optimization should be considered for the deep learning models, in order to more rigorously assess the influence of learning rate, hidden size, number of recurrent layers, and dropout values on model performance. For the Random Forest model, future optimization should focus on resource-aware model selection, including smaller ensembles, shallower trees, pruning strategies, and compression techniques to reduce storage and memory requirements while preserving predictive performance. Integration with continuous monitoring systems and prospective clinical validation will be essential steps toward translating these methods into practical tools for large-scale arrhythmia screening.

## 7 Conclusion

These results show that both feature-based and end-to-end multimodal temporal modeling strategies can effectively support multi-class arrhythmia detection from real-world smartwatch photoplethysmography and accelerometer signals. The findings highlight a clear trade-off between interpretability, overall performance, class-balanced sensitivity, and deployment feasibility: the Random Forest achieved the strongest average accuracy and macro F_1_ performance under two-fold subject-independent cross-validation, while the CNN–BiLSTM provided the highest macro recall and improved sensitivity to PAC/PVC events. Overall, the results support the feasibility of deploying artificial intelligence–based systems for continuous, near-real-time cardiac rhythm monitoring using two-channel photoplethysmography and accelerometer waveforms acquired by consumer smartwatches in free-living conditions. By providing a unified comparison between feature-based and end-to-end approaches, together with improvements over selected Pulsewatch baselines reported in the literature, this work contributes toward the development of scalable, non-invasive solutions for early arrhythmia detection and long-term cardiovascular monitoring in everyday settings.

## Data Availability

The Pulsewatch dataset analyzed in this study is available subject to approval from the data provider. Instructions for requesting access to the dataset and completing the download process are available through Synapse. Requests to access these datasets should be directed to https://www.synapse.org/Synapse:syn23565056/wiki/608635.
